# New Validated Method for the Determination of Six Opium Alkaloids in Poppy Seed-Containing Bakery Products by High-Performance Liquid Chromatography-Tandem Mass Spectrometry after Magnetic Solid-Phase Extraction

**DOI:** 10.1021/acs.jafc.2c01664

**Published:** 2022-06-08

**Authors:** Gema Casado-Hidalgo, Gonzalo Martínez-García, Sonia Morante-Zarcero, Damián Pérez-Quintanilla, Isabel Sierra

**Affiliations:** Departamento de Tecnología Química y Ambiental, ESCET, Universidad Rey Juan Carlos, C/ Tulipán s/n, 28933 Móstoles, Madrid, Spain

**Keywords:** opium alkaloids, bakery products with poppy seeds, magnetic solid-phase extraction, validation, liquid chromatography-tandem mass spectrometry

## Abstract

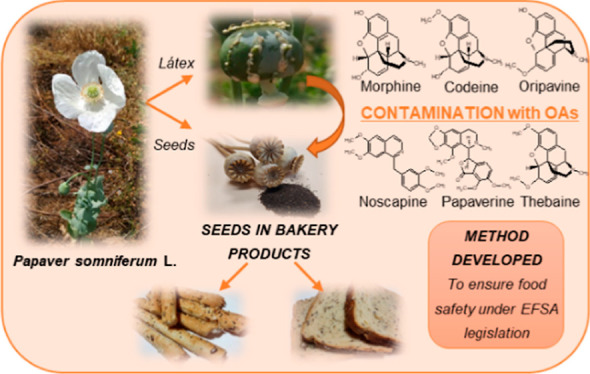

Bakery products containing poppy seeds are increasingly being commercialized. These seeds may be contaminated with latex from the *Papaver somniferum* L. plant rich in opium alkaloids (OAs). Therefore, health authorities demand the development of analytical methods to control them. In this study, an efficient and simple method was developed and validated for the first time to analyze six OAs in bakery products by high-performance liquid chromatography-tandem mass spectrometry. For this purpose, a solid–liquid extraction was optimized, and then a magnetic material [magnetite surface-modified with Fe(III) terephthalate, denoted as Fe_3_O_4_@TPA–Fe] was used for a fast magnetic solid-phase extraction. The method has been validated with adequate recoveries (70–110%) and relative standard deviations (<20%) and without matrix effects. Nine bakery samples (five breadsticks and four sliced bread) were analyzed; breadsticks showed low amounts of OAs, but two sliced bread showed higher amounts of OAs than the new amount (1.5 mg/kg) set by the Commission Regulation (EU) 2021/2142.

## Introduction

The seeds of the *Papaver somniferum* L. plant, commonly known as opium poppy, are increasingly used in bakery products (bread, buns, and biscuits), as a topping for salads or yoghurts, or in the elaboration of tea and oil. The most traded food items with poppy seeds are bakery products, mainly breadsticks and sliced bread.^1−4^ Poppy seeds hardly contain opium alkaloids (OAs), but they can be contaminated due to harvesting practices or insect damage with the OAs present in the latex of this plant (morphine, codeine, thebaine, papaverine, noscapine, and oripavine).^5,6^ Its consumption can lead to false-positive drug tests and cause adverse health effects, including cases of intoxication.^5−7^

The European Commission has published on 3 December 2021 the Regulation (EU) 2021/2142, which comes into application on 1 July 2022. This regulation sets maximum levels for OAs, expressed in morphine equivalents (morphine + 0.2 codeine) for bakery products (1.5 mg/kg) and for whole, ground, or milled poppy seeds (20 mg/kg). Furthermore, it is claimed that these levels should be set considering that food processing may reduce the OA content of raw poppy seeds by 25–100% in the final product. In this regard, the suppliers of poppy seeds should provide the morphine equivalent content of the seeds used as an ingredient to the manufacturers of bakery products.^9^ Besides, the European Commission in 2014 published recommendations for good agricultural and seed processing practices to reduce the morphine content,^10^ and in several articles, it has been published that washing, grinding, and baking treatments can decrease the content of OAs.^4,7,11^ Furthermore, in 2018, the European Food Safety Authority (EFSA) and the German Federal Institute of Risk Assessment claimed new effective analytical methods to quantify all main OAs (such as thebaine, papaverine, noscapine, and oripavine), not only morphine and codeine as in previous studies, and thus be able to legislate because they can be even more toxic as declared by health authorities and some recent studies,^12−14^ such as the review by Eisenreich et al. 2020 where the high toxicity of thebaine is reported.^8^ Considering that these compounds are found at low concentrations in very complex food matrices, analytical methods based on sensitive and selective analytical techniques are essential. The most used technique is high-performance liquid chromatography (HPLC) with mass spectrometry (MS) as recommended by the EFSA. This technique is equipped with a triple quadrupole (TQ) detector with electrospray ionization in the positive mode (ESI+) and multiple reaction monitoring (MRM) for multiple analyte detection.^3,4,6,15−17^ Regarding sample treatment, until now, solid–liquid extraction (SLE) of OAs from poppy seeds has been performed in most studies.^3,4,18,19^ However, it is essential to carry out an adequate sample treatment that includes a preconcentration and/or purification step to eliminate the possible matrix effects, thus avoiding erroneous results^3^ and extending the useful life of the chromatographic column and MS detector. For this reason, a solid-phase extraction (SPE) step is used in some studies.^15,17,20−23^ In addition, the magnetic dispersive SPE (MSPE) version has also been evaluated for this task^6,24−28^ as the sorbent material can be quickly separated from the solution by using an external magnetic field, instead of filtration or high-speed centrifugation as required in common dispersive SPE. Then, MSPE is a simpler, faster, easily miniaturized, and environmentally friendly preconcentration/purification technique.^24^ The most widely used magnetic nanoparticles consist of a magnetite (Fe_3_O_4_) core, and adding a layer of silica is popular.^6,25,26^ However, these materials only offer hydrogen bonding interactions, so alternative functionalizations are explored to achieve other types of interactions (such as π–π electrostatic and ion–dipole) that improve the interaction with OAs, by attaching a ligand either to the silica or directly to the magnetite.

The aim of this work is to develop an efficient, rapid, and very simple method to quantify six OAs in bakery products. For this purpose, a novel magnetic material composed of a magnetite surface modified with Fe(III) terephthalate (Fe_3_O_4_@TPA–Fe) was synthesized and evaluated as a sorbent. Then, an SLE–MSPE sample treatment procedure was optimized and successfully validated to apply for the quantification of six OAs in sliced bread and breadsticks by HPLC–MS/MS.

## Materials and Methods

### Reagents and Materials

Standards of morphine, codeine, thebaine, and oripavine were received from Alcaliber S.A.U. (Madrid, Spain). Noscapine, papaverine, and morphine-*d*_3_ (internal standard, IS) were obtained from Sigma-Aldrich (Zwijndrecht, The Netherlands). Individual stock standard solutions were prepared at 1000 μg/mL in methanol, and working standard solutions were prepared at 1 μg/mL in water/acetonitrile 90/10 (v/v) with 0.1% formic acid. All of these were stored in the dark at −20 °C. Ferric chloride 6-hydrate (FeCl_3_·6H_2_O) 99% and ferrous chloride 4-hydrate (FeCl_2_·4H_2_O) 99% were purchased from Labkem (Barcelona, Spain) and Acros Organics (Geel, Belgium), respectively. Terephthalic acid (TPA) was obtained from Análisis Vinicos S.L. (Ciudad Real, Spain). Ethanol absolute, formic acid (98%), and ammonia 32% (w/w) were purchased from Scharlab (Barcelona, Spain). *n*-Hexane and *N*,*N*-dimethylformamide (DMF) were purchased from Merck (Darmstadt, Germany). Acetonitrile and methanol used were of HPLC–MS quality and were purchased from Scharlab (Barcelona, Spain). Ultrapure water (resistivity 18.2 MΩ cm) was obtained from the Milli-Q water purification system (Millipore, Billerica, MA, USA). The Nd–Fe–B magnet (5 × 5 × 2 cm) with 200 kg force used in the MSPE procedure was obtained from Superimanes S.L. (Sevilla, Spain).

### Bakery Samples

In the middle of 2021, four different brands of sliced bread and five breadsticks samples were purchased from supermarkets in Madrid and Zaragoza (Spain). From each sample, three packets were taken to obtain a more representative sample as the OA content of poppy seeds can be very variable even from the same batch.^6^ The poppy seed content of these bakery products was in the range of 1–6% (Table S1). To obtain a representative and homogeneous sample with a small particle size, three packets of each sample were ground with a manual mortar so as not to grind the poppy seeds and reduce the OA levels. To facilitate grinding, sliced bread samples were frozen with liquid nitrogen, and all the samples were sieved through a pore size of 1 mm. Later, the three packets were homogenized to obtain a more representative sample. Then, sliced bread was stored at −20 °C until further analysis, and breadsticks were stored at room temperature for their longer shelf life.

### Preparation of the Fe_3_O_4_@TPA–Fe Material

First, Fe_3_O_4_ nanoparticles were prepared by chemical co-precipitation according to the work of Zhang and Shi.^29^ To do this, 15 mmol of FeCl_3_·6H_2_O and 10 mmol of FeCl_2_·4H_2_O were dissolved in 80 mL of degas ultrapure water with stirring at 300 rpm and 80 °C under a nitrogen atmosphere. Then, 50 mL of ammonia solution (32%, v/v) was added, and the mixture was stirred for 30 min. The black precipitate obtained (Fe_3_O_4_ nanoparticles) was collected with the help of a strong magnet and washed several times with deionized water until it reaches neutral pH. Finally, Fe_3_O_4_ particles were dried under vacuum by a vacuum line at 60 °C for 24 h.

For Fe_3_O_4_@TPA–Fe synthesis, 1 g of magnetic particles was mixed with 25 mL of 0.84 M FeCl_3_·6H_2_O solution in DMF through ultrasound (US) (Elmasonic S30, Elma, Singen, Germany) for 10 min. Then, 50 mL of 0.12 M TPA solution in DMF was added and subjected to US for 10 min. The mixture was placed in a 530 mL Teflon-coated stainless steel reactor (V 1.0 L, PS 131 bar, Parr Instrument Company, Moline, Illinois, USA) and maintained at 100 °C for 10 h. The final product (2 g) was collected using a magnet, washed with hot ethanol, and dried under vacuum at 60 °C for 24 h. The TPA–Fe material was also synthesized in a similar way.

### Characterization of the Fe_3_O_4_@TPA–Fe Material

The synthetized material was characterized by scanning electron microscopy (SEM), transmission electron microscopy (TEM), attenuated total reflection-Fourier transform infrared (ATR-FTIR) spectroscopy, powder X-ray diffraction (XRD), N_2_ gas adsorption–desorption isotherms, and elemental analysis. Details of the equipment and conditions can be found in Supporting Information S1.

### Study of Adsorption and Desorption Conditions on the Fe_3_O_4_@TPA–Fe Material with Standards

First, the adsorption was optimized. To do this, the studies were performed in duplicate and with a standard solution of 1 μg/mL of each of the six OAs. The parameters evaluated were of solvent type (methanol, acetonitrile, acetone, isopropanol, ethyl acetate, dichloromethane, and hexane), at different times (1, 5, 10, and 20 min), and with different amounts (the maximum expected amount is 50 mg), and then the proportion of added ammonia or formic acid (10%) was evaluated. Subsequently, different quantities of the material were studied (1, 2.5, 5, 10, 20, and 50 mg) to decrease it without affecting the adsorption. Finally, to optimize desorption, the type of the solvent (methanol, acidified methanol, water, acetonitrile, and a mixture of water/acetonitrile, 90/10, v/v, containing 0.1% acid formic) and the desorption time (1, 5, and 10 min) were evaluated.

### Optimized Bakery Sample Preparation Procedure by SLE–MSPE

First, optimization of the SLE of OAs from bakery samples was carried out. To do this, two types of extraction solvents (methanol with 0.1% acetic acid and hexane) and two sample amounts (2.5 and 5 g) were studied. For this, a double SLE was performed with 10 mL for 30 min under magnetic stirring according to the conditions previously used by other authors in the literature^4^ and in our previous work.^6^ To select the best conditions, recoveries obtained for the different parameters evaluated were compared. The values obtained for samples spiked at two concentration levels were compared with the values obtained for blank samples subjected to the same SLE process but spiked at the end, prior to HPLC–MS/MS analysis. The spiking of the samples was done considering that the average proportion of poppy seeds in the bakery samples was around 5% (as shown in Table S1). Consequently, two spiked levels were evaluated, estimating that a high amount (5 mg/kg) and a low amount (0.25 mg/kg) of OAs could be found in the sample based on our previous work in which different poppy seeds were analyzed.^6^

**Figure 1:**
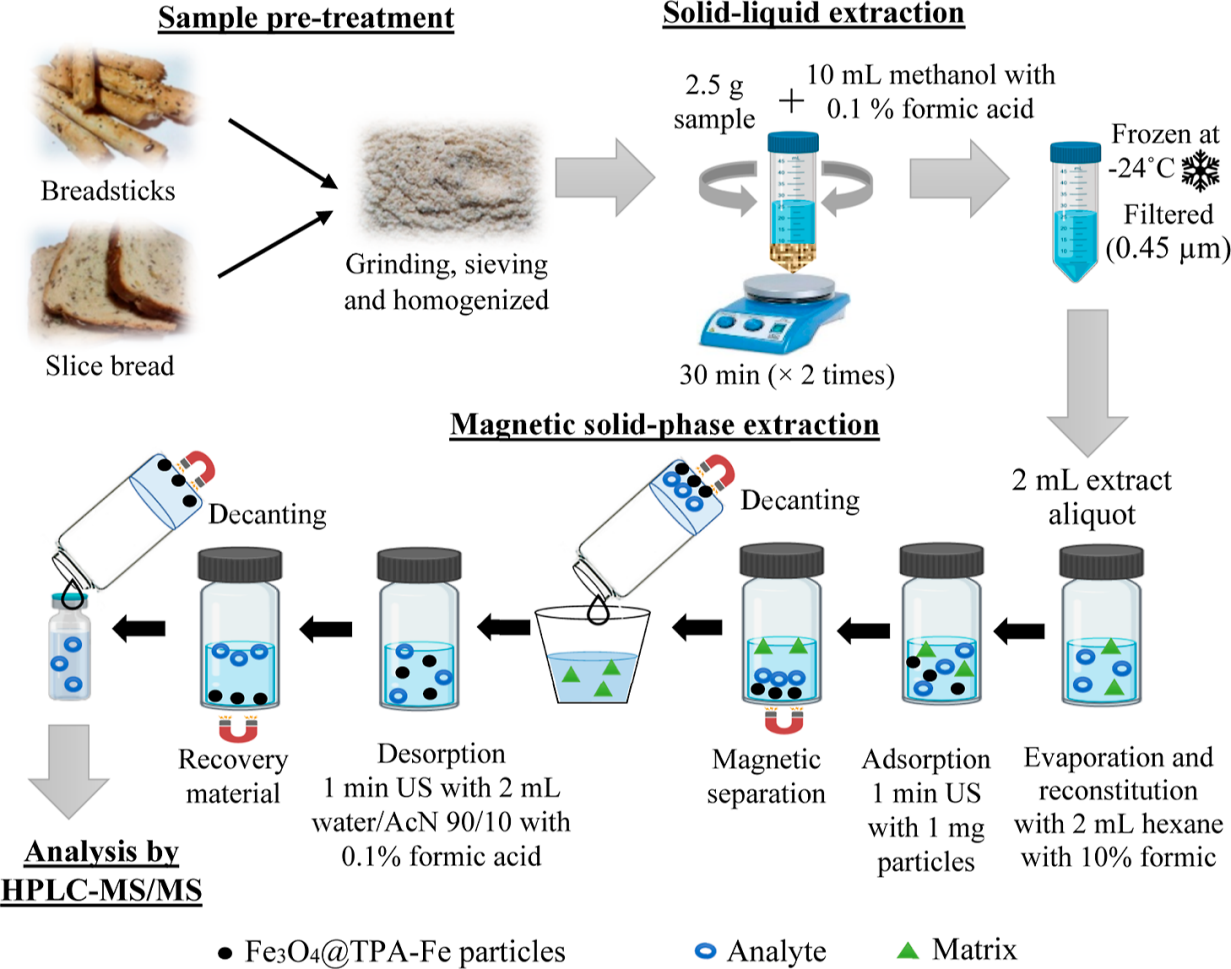
Diagram of the proposed methodology to quantify OAs in breadstick and sliced bread samples.

Once all the conditions were optimized, the method developed after grinding, homogenizing, and sieving consisted of (as shown in Figure 1) a double extraction of 2.5 g of sample with 10 mL of methanol acidified with 0.1% acetic acid for the SLE. The mixture was vortexed for 30 s (Rx^3^ Velp Scientifica, Usmate, MB, Italy) and magnetically stirred for 30 min. Later, it was centrifuged at 6000 rpm (3992 rcf) for 10 min to recover the supernatant (ROTOFIX 32A Hettich, Tuttlingen, Germany). Then, the extract was frozen at −24 °C and filtered through a 0.45 μm nylon filter to remove fats, and 2 mL of extract solution was evaporated to dryness under vacuum and reconstituted in 1 mL of acidified hexane. Next, 1 mg (weighed in an Excellence Plus XP-6 Mettler with a deviation of 1 μg) of Fe_3_O_4_@TPA–Fe (conditioned with 1 mL of acidified hexane for 1 min in the US) was added into the reconstituted extract, followed by US for 1 min. The material was separated by a magnet from the solution, and the analytes were desorbed with 2 mL of water/acetonitrile (90/10, v/v) with 0.1% formic acid for 1 min in the US. Finally, the solution was decanted for 2 min with the magnet, an aliquot of 950 μL was taken, and 50 μL of 1 μg/mL morphine-*d*_3_ (IS) was added before HPLC–MS/MS analysis (Figure 1).

### HPLC–MS/MS Analysis

Quantification of OAs in bakery products was performed with a Varian 1200/1200 LC (Varian Ibérica, Madrid, España) equipped with a ProStar 410 autosampler (100 μL loop) coupled with a TQ tandem mass spectrometer detector (1200 L TQ) with an electrospray ionization (ESI) ion source. The data acquisition system was MS Workstation Varian version 6.8. Chromatographic separation was performed as mentioned in our previous work,^6^ using a C_18_ KromaPhase 100 column (150 × 2.0 mm, 3.5 μm particle size, Scharlab, Barcelona, Spain) at 30 °C. The injection volume was 10 μL (partial injection), and the flow rate was set at 0.25 mL/min. A gradient elution similar to our previous work^6^ was used with a mobile phase of water (A) and acetonitrile (B), both with 0.1% of formic acid as follows: 90–30% A (0–6 min), 30–90% A (6–9 min), and 90% A (9–11 min) for column re-equilibration. Mass spectrometry acquisition was performed with electrospray ionization in the positive mode (ESI+) with the MRM mode. N_2_ was used as the drying and nebulizer gas. The frying gas was set at 350 °C and 22 psi, and the nebulizer gas was set at 58 psi. The capillary voltage was held at 5000 V and shielded at 600 V. Argon was used as the collision gas at 1.90 mTorr and a detector voltage of 1480 V. The detection of each analyte was performed by direct infusion of a standard solution of 1 μg/mL in methanol using a syringe pump at a flow rate of 20 μL/min. The mass peak width in *Q*_1_ is 2.5, the mass speak width in *Q*_3_ is 2.5, and the scan width in MRM is 2 s.

### Method Validation

The methodology was validated for analyzing breadsticks and sliced bread because although they are bakery products, they are relatively different samples. This was done by following the SANTE/12682/2019 document^35^ since there is currently no official regulation on analytical performance requirements for OAs in food or feed. The validation was done in terms of linearity, method detection limits (MDLs), quantification limits (MQLs), matrix effect (ME), accuracy, precision, and selectivity (more details in Supporting Information S2).

## Results and Discussion

### Preparation and Characterization of the Fe_3_O_4_@TPA–Fe Material

The SEM images of Fe_3_O_4_ (Figure S1a) and Fe_3_O_4_@TPA–Fe (Figure S1b) showed small spherical particles, with a tendency to aggregate, which is very common in magnetic materials. The TEM images suggested that F_3_O_4_@TPA–Fe particles (Figure S1c,d) were assembled on each other in 3D network macroporous structures with an average size of around 300 × 700 nm.

The FTIR spectra of Fe_3_O_4_, TPA, Fe–TPA, and Fe_3_O_4_@TPA–Fe are shown in Figure S2. The band at 520–530 cm^–1^ can be assigned to the Fe–O bond stress, which is observed in Fe_3_O_4_, TPA–Fe and Fe_3_O_4_@TPA–Fe. The signals between 3200 and 3500 cm^–1^ are the stretching bands of the −OH groups on the surface of the magnetite as the functionalization with the TPA–Fe compound decreases a lot, indicating the interaction between TPA–Fe and Fe_3_O_4_. The FTIR spectrum of TPA shows the characteristic bands of this organic compound at 925, 1272, and 1417 cm^–1^, corresponding to the bending bands of the carboxylate group (COO^–^) and the stress band of the carbonyl group (C=O), which appear at around 1680 cm^–1^. The carbonyl signal at around 1680 cm^–1^ in TPA practically disappears in the TPA–Fe compound because of the interaction with the Fe atoms. Between 3000 and 2500 cm^–1^ appear the stretching bands corresponding to the carboxylate group (COO^–^). The FTIR spectrum of the Fe_3_O_4_@TPA–Fe particles also shows the characteristic bands of the carbonyl group of TPA at 1291 and 1634 cm^–1^ corresponding to the stretching of the C=O group, and the stretching bands of the carboxylic acid functional group (COO^–^) are also observed around 3040–3116 cm^–1^.^36^ Therefore, FTIR analysis confirmed the interaction between the TPA–Fe compound and the surface of Fe_3_O_4_.

The XRD pattern (Figure S3) of Fe_3_O_4_ and Fe_3_O_4_@TPA–Fe agreed with the theoretical pattern of Fe_3_O_4_ described in the bibliography.^37^ There are six discernible diffraction peaks in the 2θ region of 20–70° (220, 311, 400, 422, 511, and 440) that correspond to the Miller index diffraction peaks (JCPDS card: 19-0629), showing that the magnetite core is still present after modification. The size of particles was calculated using Scherrer eq 1
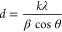
1where *k* is a constant (*k* = 0.9), λ is the wavelength of X-rays (1.5418 Å), β is the full width at half-maxima of the diffraction peak line (in radians), and θ is the half of the diffraction angle. Fe_3_O_4_ was estimated to have a size of ∼9 nm and Fe_3_O_4_@TPA–Fe to be of ∼13 nm.

In addition, N_2_ gas adsorption–desorption isotherms were made. Fe_3_O_4_ presents a type IV isotherm according to the IUPAC classification^38^ (Figure S4a). As can be seen in Table S2, the surface area of Fe_3_O_4_ is 105 m^2^/g, the pore volume of Fe_3_O_4_ is 0.30 cm^3^/g, and the pore distribution of Fe_3_O_4_ is at 41.3 Å according to other studies with the chemical co-precipitation method.^39^ The pore diameter that appears at 130.1 Å corresponds to the inter-particle space; this phenomenon is also observed in other porous materials that can give rise to particle agglomerates or overlapping layers of the material.^40^ This coincides with the type of hysteresis, which is of type H1, typical of agglomerates as can be observed in the SEM image (Figure S1a). Fe_3_O_4_@TPA–Fe presented a type II isotherm with a H1 hysteresis (Figure S4b). In this case, the surface area and pore volume were lower (47 m^2^/g and 0.14 cm^3^/g, respectively), showing the correct functionalization of the Fe_3_O_4_ particles. Moreover, the pore distributions obtained were 21.0 and 93.3 Å, corresponding to the pores in TPA–Fe, and 131.8, 237.8, and 488.3 Å, corresponding to the inter-particle spaces between the Fe_3_O_4_ particles (Table S2), which present an irregular distribution as shown in the TEM images (Figure S1c,d).

Finally, the % C calculated by elemental analysis was around 3%, and the functionalization degree estimated was 0.31 mmol TPA/g of material and the % N was 0% N, which confirms the complete elimination of the synthesis solvent (DMF).

### Study of Adsorption and Desorption Conditions on the Fe_3_O_4_@TPA–Fe Material with Standards

**Figure 2:**
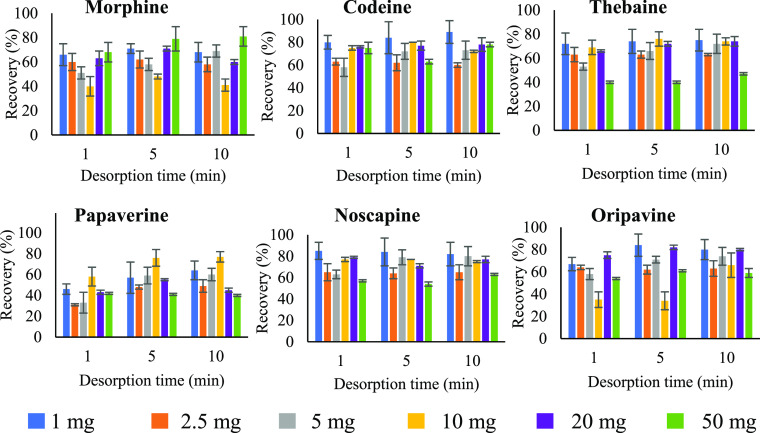
Comparison of the recovery (%) between different amounts of the Fe_3_O_4_@TPA–Fe material (1, 2.5, 5, 10, 20, and 50 mg) in 2 mL of hexane with 10% formic acid with 1 μg/mL of each of the six analytes during 1 min adsorption and different desorption times (1, 5, and 10 min).

**Figure 3:**
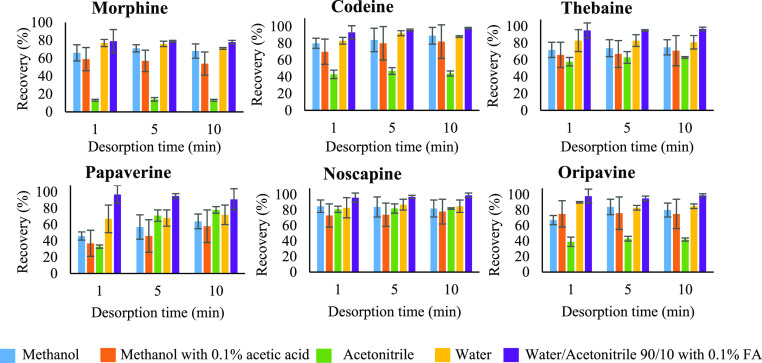
Comparison of the recovery (%) between different desorption solvents with 1 mg of Fe_3_O_4_@TPA–Fe material in 2 mL of hexane with 10% formic acid with 1 μg/mL of each of the six analytes during 1 min adsorption and different desorption times (1, 5, and 10 min).

The adsorption solvent was first determined to ensure the highest adsorption of the analytes. For this purpose, 2 mL of a 1 μg/mL solution of each of the six OAs in solvents of different polarities (methanol, acetonitrile, acetone, isopropanol, ethyl acetate, dichloromethane, and hexane) was mixed with 50 mg of Fe_3_O_4_@TPA–Fe material through US for 1, 5, 10, and 20 min, and the supernatants were analyzed. As shown in Table 1, different behaviors were observed depending on the analytes. For morphine, codeine, and oripavine, high adsorptions were obtained with all solvents except methanol. However, thebaine, papaverine, and noscapine only showed high adsorption with hexane, so it was the solvent selected for adsorption. Besides, all the analytes were completely adsorbed in 1 min, except noscapine which showed its maximum adsorption percentage (98%) after 20 min. Later, the addition of formic acid or ammonia was evaluated, so adsorption values were calculated after different times (1, 5, 10, 20, 30, and 60 min) in hexane, with 10% formic acid and 10% ammonia. As can be seen in Figure S5, with hexane with 10% ammonia, thebaine, papaverine, and noscapine adsorption were nearly 0%. However, hexane with 10% formic acid showed the best adsorption for noscapine after 1 min. Once adsorption has been optimized, the influence of the amount of material on the recovery of the analytes was also studied. For this task, different amounts (1, 2.5, 5, 10, 20, and 50 mg) were mixed for 1 min with 2 mL of a 1 μg/mL solution in acidified hexane of each of the six OAs. Desorption was carried out with 2 mL of methanol for 1, 5, and 10 min. As shown in Figure 2, amounts higher than 1 mg showed invariant recoveries. Therefore, 1 mg of Fe_3_O_4_@TPA–Fe material was selected as the optimized adsorbent amount for the MSPE procedure. Subsequently, 2 mL of different types of desorption solvents (methanol, methanol with 0.1% acetic acid, acetonitrile, water, and water/acetonitrile, 90/10, v/v, with 0.1% formic acid) was tested. As shown in Figure 3, the best recovery values were achieved with water/acetonitrile (90/10, v/v) with 0.1% formic acid in 1 min. Therefore, 2 mL of water/acetonitrile (90/10, v/v) with 0.1% formic acid and 1 min were selected as the optimum desorption conditions. Finally, the whole MSPE procedure developed was evaluated under optimized conditions using 1 mg of Fe_3_O_4_ and, as it was expected, recoveries were nearly 0%. These results highlight the role of TPA–Fe in the adsorption of the target analytes. Figure S6 and Supporting Information S3 show a proposal of possible molecular interactions that can occur between the adsorbent material and the target analytes (e.g., with morphine).

**Table 1:** Adsorption Percentages (%) ± Standard Deviation (SD) Obtained for Each of the OAs with Seven Types of Solvents for Different Times with Fe_3_O_4_@TPA–Fe Material^a^

adsorption solvent	adsorption time (min)	morphine	codeine	thebaine	papaverine	noscapine	oripavine
AcN	1	98 ± 3	60 ± 3	52 ± 0	49 ± 0	20 ± 1	90 ± 2
	5	98 ± 1	60 ± 1	52 ± 2	46 ± 2	19 ± 2	91 ± 1
	10	98 ± 2	67 ± 4	52 ± 1	44 ± 1	19 ± 0	92 ± 3
	20	98 ± 2	68 ± 2	50 ± 3	42 ± 3	20 ± 3	92 ± 2
MeOH	1	39 ± 4	38 ± 4	33 ± 4	20 ± 2	19 ± 2	35 ± 3
	5	32 ± 2	30 ± 4	24 ± 3	14 ± 4	13 ± 2	28 ± 4
	10	30 ± 10	24 ± 7	18 ± 1	12 ± 1	10 ± 3	18 ± 6
	20	13 ± 7	11 ± 4	6 ± 2	2 ± 1	0 ± 0	14 ± 3
DCM	1	0 ± 1	30 ± 5	2 ± 1	30 ± 5	28 ± 2	60 ± 5
	5	100 ± 0	72 ± 3	46 ± 3	45 ± 4	46 ± 7	70 ± 1
	10	100 ± 0	63 ± 4	43 ± 4	42 ± 2	42 ± 2	72 ± 1
	20	100 ± 0	80 ± 5	45 ± 1	44 ± 1	42 ± 1	85 ± 3
EtOAc	1	94 ± 2	72 ± 9	66 ± 1	16 ± 1	13 ± 2	84 ± 1
	5	95 ± 1	72 ± 3	67 ± 2	18 ± 1	15 ± 1	86 ± 1
	10	95 ± 2	70 ± 2	67 ± 1	19 ± 1	17 ± 2	88 ± 1
	20	95 ± 2	71 ± 3	66 ± 1	20 ± 2	15 ± 1	88 ± 3
IPOH	1	76 ± 3	44 ± 10	53 ± 1	32 ± 1	5 ± 1	76 ± 1
	5	77 ± 1	54 ± 9	60 ± 1	43 ± 1	11 ± 1	88 ± 0
	10	84 ± 2	56 ± 3	62 ± 1	44 ± 1	13 ± 1	89 ± 2
	20	85 ± 3	53 ± 3	59 ± 0	41 ± 0	12 ± 2	92 ± 2
Hx	1	100 ± 0	100 ± 0	99 ± 0	98 ± 1	60 ± 8	100 ± 0
	5	100 ± 0	100 ± 0	100 ± 0	99 ± 1	80 ± 10	100 ± 0
	10	100 ± 0	100 ± 0	100 ± 0	100 ± 0	93 ± 5	100 ± 0
	20	100 ± 0	100 ± 0	100 ± 0	100 ± 0	98 ± 1	100 ± 0
Ace	1	97 ± 1	76 ± 7	68 ± 7	29 ± 16	27 ± 15	91 ± 3
	5	98 ± 1	78 ± 8	69 ± 7	28 ± 15	14 ± 14	93 ± 2
	10	98 ± 2	76 ± 7	67 ± 7	26 ± 17	13 ± 13	95 ± 2
	20	99 ± 1	75 ± 6	67 ± 7	22 ± 17	20 ± 17	95 ± 1

a AcN: acetonitrile; MeOH: methanol; DCM: dichloromethane; EtOAc: ethyl acetate; IPOH: isopropanol; Hex: hexane; and Ace: acetone.

### Adsorption Kinetics and Isotherm Experiments with the Fe_3_O_4_@TPA–Fe Material

To study the adsorption kinetics, 1 mg of material was added to 2 mL of hexane with 10% formic acid with each of the six OAs (1 μg/mL) and through US at different times (1, 5, 10, and 20 min). After reaching the equilibrium, aliquots of the supernatant were analyzed by HPLC–MS/MS. The adsorption capacity (*Q*_e_) was calculated by eq 1 in Table S3, and the adsorption kinetics were determined by Lagergren’s pseudo-first order,^30^ pseudo-second order,^31^ and intra-particle diffusion kinetic models^32^ (Table S3). As shown in Figure S7a, the adsorption of all analytes is very fast because in 1 min, 100% adsorption was obtained and remained constant in the following time. In addition, the important results of the three kinetic models were compiled in Table S4 and Figure S8a. The linear regression coefficients (*R*^2^) more close to 1 in the pseudo-second-order model and their *Q*_e,cal_ (calculated result) were more similar to *Q*_e,exp_ (experimental result), showing a chemical adsorption mechanism. Besides, all compounds did not show intra-particle diffusion tendency, as their *R*^2^ values were much lower than 1.

For adsorption isotherms, 2 mL solutions of different concentrations of the six OAs (0.01, 0.1, 1, 10, 20, 30, and 40 μg/mL) were added to 1 mg of the material and 1 min US was applied. After reaching the equilibrium, aliquots of the supernatant were analyzed by HPLC–MS/MS and determined by Langmuir^33^ and Freundlich^34^ models (Table S3). As shown in Figure S7b, by increasing the initial OA concentration, the adsorption capacity was increased until the last point where the adsorption capacity of all analytes remains constant. In addition, the *R*^2^ value obtained by the Freundlich model was closer to 1 than that obtained by Langmuir, especially for thebaine, papaverine, and noscapine (Figure S8b).

### Optimization of SLE of OAs from Bakery Products

To optimize the SLE, two types of extraction solvents (methanol with 0.1% acetic acid and hexane) and two sample amounts (2.5 and 5 g) were studied. For this, a double SLE was performed with 10 mL for 30 min under magnetic stirring to ensure a complete extraction. Optimization studies were performed with sliced bread and breadsticks samples at two concentration levels, high (5 mg/kg) and low (0.25 mg/kg). First, the solvent type was evaluated. To do this, 2.5 g of each sample was extracted with 10 mL of acidified hexane (×2). This solvent was tested, as it was the best adsorption solvent for the MSPE procedure. However, the recovery values obtained did not exceed 2% for any of the analytes. Therefore, a different solvent had to be used, and consequently, a vacuum evaporation step had to be introduced between the SLE and the MSPE procedures. In this regard, the most widely used solvents to extract OAs from poppy seeds or poppy seed food products are methanol with 0.1% acetic acid^4^ and acetonitrile/water/formic acid (80/19/1, v/v/v).^3,41^ Considering that methanol with 0.1% acetic acid would be evaporated easily, this solvent was tested for the extraction of the alkaloids from the poppy seed-containing bakery samples. Results suggested that this solvent provided a good extraction efficiency due to the polarity and miscibility of the alkaloids in acidified polar solvents. Thus, satisfactory recovery values were obtained for all the analytes at the two concentration levels, being 81–102 and 99–110% for the high level and 94–121 and 82–93% for the low level in breadsticks and sliced bread samples, respectively. Furthermore, an additional study at the higher concentration level was performed with 5 g of sample but using the same amount of extraction solvent and extraction time. However, in this study, the recovery values obtained were lower, 68–89% for the sliced bread sample and 86–95% with breadsticks. For this reason, the sample amount selected for the studies was 2.5 g because it was enough to quantify the analytes at the low spiking level.

### Optimization of HPLC–MS/MS Analysis

The parameters were optimized for the OAs with electrospray ionization in the positive mode (ESI+). To do this, a 1 μg/mL methanol standard solution of each analyte was directly infused through a syringe pump at 20.0 μL/min. First, the molecular ion was detected with a *Q*_1_ resolution of 0.7 at a scan time of 500 ms and, to obtain the maximum fragment ion intensity, the collision energy was optimized such as shown in Table S5. For chromatographic separation, different mobile phases were evaluated. Water containing 0.1% formic acid was used as eluent A and acidified acetonitrile or methanol (with 0.1% of formic acid) as eluent B. Finally, higher peak intensities and better separation were obtained with acidified acetonitrile. Besides, different gradients were tested, starting with a higher proportion of water at the beginning and increasing the organic phase, depending on the retention time, and longer ramps were made. Finally, the selected gradient was 90% A (at 0 min), 30% A (at 6 min), and 90% A (at 9 to 11 min). The retention time obtained for each analyte is shown in Table S5.

Standard working solutions were analyzed by HPLC–MS/MS to evaluate the instrumental parameters. Results are shown in Table S6. Linearity was evaluated in a 0.001–1 μg/mL range for thebaine, papaverine, and noscapine and the 0.01–1 μg/mL range for morphine, codeine, and oripavine, with *R*^2^ ≥ 0.999. As can be seen, low LOD and LOQ values were obtained, between 0.06 (noscapine) and 1.5 (codeine) μg/L and between 0.1 (papaverine and noscapine) and 6 (oripavine) μg/L, respectively.

### Method Validation

**Figure 4:**
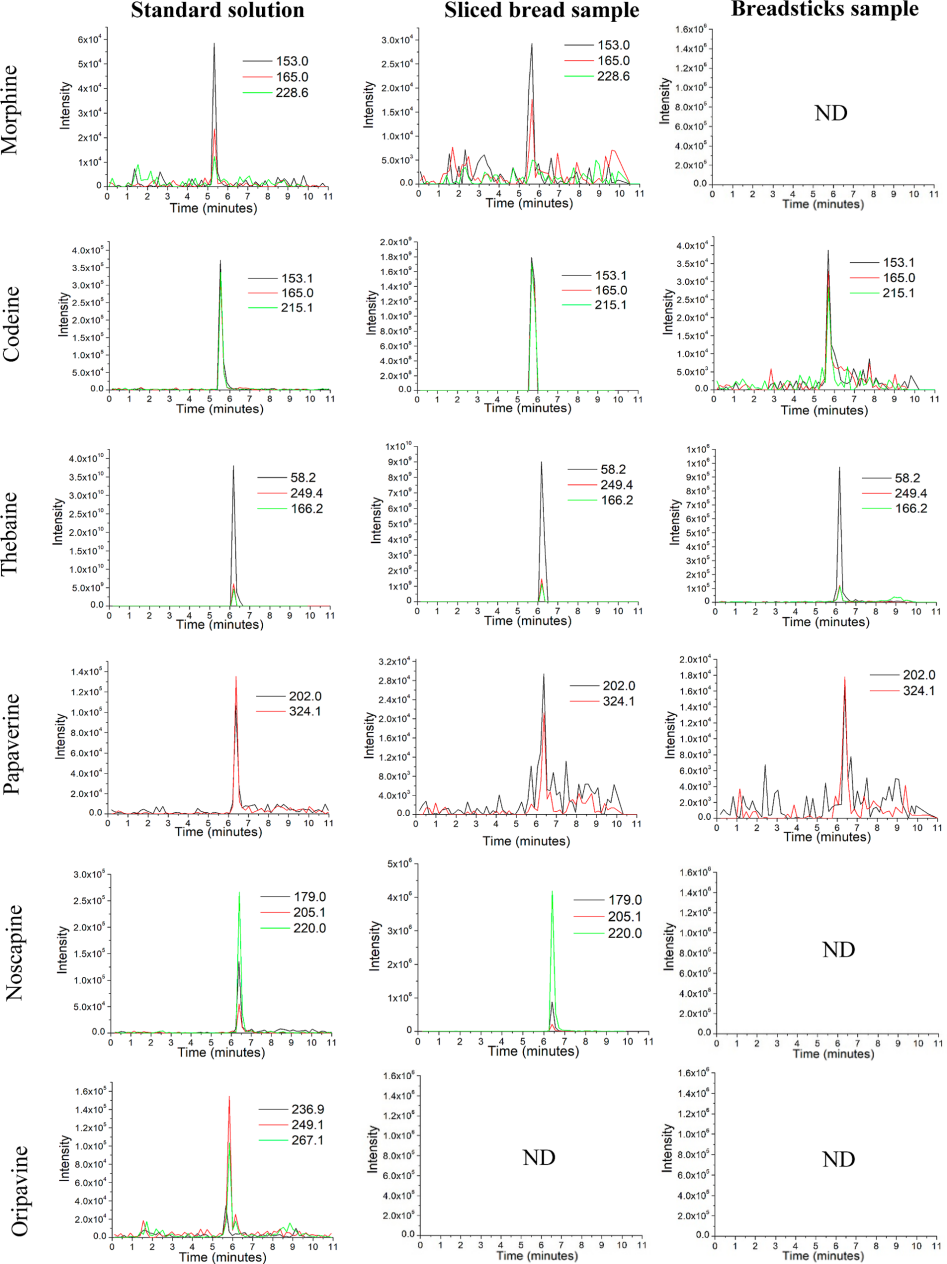
Comparison between the extracted ion chromatograms obtained for each of the OAs in a standard solution mixture of 0.001 μg/mL (thebaine, papaverine, and noscapine) and 0.01 μg/mL (morphine, codeine, and oripavine) with respect to the extracts of sliced bread and breadsticks. ND: not detected.

The validation results of the proposed SLE–MSPE-HPLC–MS/MS method for the quantification of six OAs in breadsticks and sliced bread samples are shown in Table 2. Calibration lines with *R*^2^ between 0.999 and 1.000 were obtained, and the deviation of the back-calculated concentrations of the calibration standards from the true concentrations in the matrix calibration lines was −7 and −19% for breadsticks and −0.7 and −20% for sliced bread. Therefore, these results demonstrated the good linearity of the method, which states good linearity when the deviation of the back-calculated concentrations is ≤±20%.^35^ In addition, the deviation of the slopes of the calibration lines for different days (*n* = 3) was calculated to ensure their reproducibility, obtaining RSDs between 3 and 8% in the case of breadsticks and between 1 and 9% in the case of sliced bread. On the other hand, ME was calculated by comparing the slopes of both matrix-matched and solvent-based calibration curves. ME was not observed in the breadsticks samples (<±20%), and in the sliced bread samples, a slight signal suppression was observed for thebaine, papaverine, and oripavine, being ME −36, −22, and −25%, respectively (Table 2). This means that the developed purification procedure was able to eliminate almost all possible matrix effects for the six target analytes in both bakery samples. MDL and MQL values were low for the two sample matrices. For the breadsticks samples, the MDL and MQL obtained were 1.3 and 5 μg/kg for noscapine, 1.6 and 5 μg/kg for thebaine, 3 and 8 μg/kg for papaverine, 6 and 20 μg/kg for morphine, and 13 and 42 μg/kg for codeine and oripavine, respectively. For sliced bread, the MDL and MQL obtained were 0.3 and 1 μg/kg for thebaine and noscapine, 0.5 and 1.5 μg/kg for papaverine, 2 and 7 μg/kg for codeine and morphine, and 12 and 40 μg/kg for oripavine, respectively. The accuracy and precision were evaluated at two different levels of concentration, low (0.25 mg/kg) and high (5 mg/kg), showing adequate recovery values in both samples, between 70 and 120% (Table 2). On the other hand, as shown in Table 2, satisfactory results were obtained for intra-day and inter-day precision at two concentration levels because the RSD values were lower than 20%. Furthermore, as shown in Figure 4, a good selectivity of the method was obtained. The chromatograms of the extracted ions obtained for each of the OAs in a standard solution were compared with the extracts of each sample. It was obtained that the variation of *t*_R_ was ≤0.1 min, and the ion ratios of the sample extracts were within ±30% (relative abundance) of the mean of the standards for each analyte.

**Table 2:** Validation Parameters of the SLE–MSPE-HPLC–MS/MS Method for the Quantification of Six OAs in Bakery Products^a^

						accuracy^f^		precision^f^	
analytes	linear range (μg/mL)	matrix-matched calibration (*R*^2^)^b^	ME^c^	MDL^d^ (μg/kg)	MQL^e^ (μg/kg)	recovery (% ± SD)	mean recovery (% ± SD)	intra-day precision (RSD %)	inter-day precision (RSD %)
Method Validation with Breadsticks Samples									
morphine	0.01–1	*y* = 3.2 × 10^6^*x* + 1.2 × 10^5^ (0.999)	–11	6	20	71 ± 9^L^	77 ± 6	17^L^	20^L^
						83 ± 3^H^		4^H^	9^H^
codeine	0.01–1	*y* = 4.1 × 10^6^*x* + 7.0 × 10^4^ (1.000)	–7	13	42	75 ± 8^L^	80 ± 6	13^L^	18^L^
						84 ± 3^H^		4^H^	5^H^
thebaine	0.001–1	*y* = 2.7 × 10^7^*x* + 4.8 × 10^5^ (1.000)	–36	1.6	5	88 ± 10^L^	83 ± 7	15^L^	19^L^
						77 ± 3^H^		4^H^	7^H^
papaverine	0.001–1	*y* = 4.6 × 10^7^*x* + 1.1 × 10^6^ (0.999)	–22	3	8	66 ± 11^L^	70 ± 9	19^L^	19^L^
						73 ± 7^H^		10^H^	12^H^
noscapine	0.001–1	*y* = 7.2 × 10^7^*x* + 1.2 × 10^6^ (1.000)	–11	1.3	5	79 ± 9^L^	81 ± 6	14^L^	17^L^
						83 ± 2^H^		2^H^	5^H^
oripavine	0.01–1	*y* = 4.1 × 10^6^*x* + 4.7 × 10^4^ (0.999)	–25	13	42	85 ± 9^L^	84 ± 6	14^L^	17^L^
						82 ± 3^H^		3^H^	5^H^
Method Validation with Sliced Bread									
morphine	0.001–1	*y* = 4.7 × 10^6^*x* + 9.5 × 10^3^ (1.000)	20	2	7	89 ± 9^L^	90 ± 9	10^L^	18^L^
						90 ± 9^H^		9^H^	11^H^
codeine	0.001–1	*y* = 5.2 × 10^6^*x* + 2.7 × 10^5^ (1.000)	17	2	7	66 ± 11^L^	93 ± 9	20^L^	20^L^
						120 ± 7^H^		6^H^	7^H^
thebaine	0.001–1	*y* = 3.9 × 10^7^*x* + 3.5 × 10^7^ (1.000)	**–2**	0.3	1	78 ± 10^L^	91 ± 10	17^L^	20^L^
						104 ± 9^H^		9^H^	11^H^
papaverine	0.001–1	*y* = 6.4 × 10^7^*x* + 5.3 × 10^5^ (1.000)	15	0.5	1.5	95 ± 14^L^	99 ± 11	15^L^	19^L^
						103 ± 8^H^		8^H^	9^H^
noscapine	0.001–1	*y* = 8.8 × 10^7^*x* + 1.6 × 10^6^ (1.000)	12	0.3	1	106 ± 4^L^	110 ± 7	4^L^	13^L^
						114 ± 9^H^		8^H^	12^H^
oripavine	0.01–1	*y* = 5.6 × 10^6^*x* + 5.3 × 10^4^ (1.000)	3	12	40	110 ± 10^L^	105 ± 9	9^L^	10^L^
						100 ± 8^H^		8^H^	9^H^

a Linear range expressed in μg/kg is 80–8000 in the case of morphine, codeine, and oripavine in breadsticks and in oripavine in sliced bread and 8–8000 in all other cases.

b The calibration line is in the units: μg/mL.

c ME: matrix effect (dividing the purified matrix slope by the solvent slope).

d MDL: method detection limit.

e MQL: method quantification limit.

f Accuracy and precision were obtained by spiking samples at two known concentration levels: low (L, 0.25 mg/kg) and high (H, 5 mg/kg).

### Comparison with Other Reported Methods

The proposed methodology was compared with other methods previously published (Table 3). To the best of our knowledge, this is the first validated method for the simultaneous analysis of six OAs in bakery products with poppy seeds. There are only three articles in bakery products, but their methods used were validated in poppy seeds, which are less complex matrices. In addition, a simple SLE was performed to extract the alkaloids from the bakery products, without a purification step to eliminate/reduce the possible matrix effects prior to chromatographic analysis.^2−4^ This is a very important step in the analytical process as matrix effects can cause false results and increase equipment deterioration. Three studies analyzed the hotpot seasoning samples (a Chinese popular food) with SPE and MSPE for clean-up purposes.^15,27,28^ For example, Guo et al. used 60 mg of a commercial adsorbent (Oasis MCX) for the SPE step.^13^ However, novel magnetic materials have also been evaluated for this task, looking for the reduction of sorbent quantities and time. Thus, Xu et al. used 50 mg of amantadine-functionalized magnetic microspheres (Fe_3_O_4_@SiO_2_@ADME),^28^ and Tang et al. used 15 mg of a magnetic chitosan composite material (Fe_3_O_4_@SiO_2_@CS/GO)^27^ for the MSPE step. In these protocols, the adsorption step required 8^28^ and 20 min^27^ and for desorption 2 min. Finally, in our recent work,^6^ mesostructured silica-coated magnetic nanoparticles (Fe_3_O_4_@SiO_2_@mSiO_2_) were used as an adsorbent for MSPE to purify the poppy seed sample extracts in just 4 min, but 50 mg of material was needed for this purpose. Thus, regarding these previous studies, emphasis should be put on in the current work since only 1 mg of adsorbent material is needed, and the purification step takes only 1 min for adsorption and another for desorption. Furthermore, with the Fe_3_O_4_@TPA–Fe material, the matrix effects were avoided to a greater extent for all analytes and for both sample types in contrast with other methods that reflect serious signal suppression^13,27^ or enhancement^3,15^ for some analytes. Another point to highlight is that the recoveries obtained with the Fe_3_O_4_@TPA–Fe material were all in the adequate range, and with the previous material, two of them were around 50% (morphine and oripavine) because they showed a higher intra-particle effect in the adsorption kinetics, and therefore, it was not possible to completely desorb them from the material.^6^ In addition, the analytical characteristics of the method were also compared with those of the previously reported methods for the determination of OAs but in other simpler sample matrices (Table 3). The MDL and MQL achieved with this methodology were sufficiently low for these analytes in these types of samples (0.3–13 and 1–42 μg/kg, respectively) and better or comparable accuracy (70–110%), and an adequate precision (≤20%) was also obtained. Therefore, the proposed method is a good alternative for an efficient, rapid, and simple determination of six OAs in bakery products with poppy seeds.

**Table 3:** Comparison of the Proposed Methodology with Other Methods Previously Published for the Quantification of OAs in Food^a^

			sample treatment			validation parameters					
sample analyzed	sample validation	analyte	extraction	purification	analysis technique	MDL (μg/kg)	MQL (μg/kg)	ME (%)	recovery (%)	RSD (%)	refs
poppy seeds, poppy seed topped rolls, muffins	poppy seed (200 mg)	MOR, COD, THEB, NOS, PAP	Chl/IPOH (90/10, v/v) at pH 3.5 (1 mL, 10 min)		HPLC-IT-MS/MS					≤6	(2)
poppy seeds, filling for bakery and cakes	poppy seed (10 g)	MOR, COD, THEB, NOS, PAP, NAR	AcN/water/formic acid, 80/19/1, v/v/v (100 mL, 30 min × 2)		UHPLC-TQ-MS/MS		100	100–130	77–172	≤20	(3)
poppy seeds, cakes, buns	poppy seed (10 g)	MOR, COD, PAP, NOS	MeOH 0.1% acetic acid (30 mL, 60 min)		HPLC-TQ-MS/MS	70–300	200–1000			≤9	(4)
poppy seeds	poppy seeds (2.5 g)	MOR, COD, THEB, PAP, NOS, ORIP	MeOH/water, 50/50 (v/v) (30 mL, 30 min × 2)	Fe_3_O_4_@SiO_2_@mSiO_2_ (50 mg)	UHPLC-TQ-MS/MS	0.07–72.01	0.24–240	31–109	46–109	≤11	(6)
hot pot	hot pot (5 g)	MOR, COD, THEB, PAP, NOS	HCl 0.1 M (20 mL, 10 min) and PE (10 mL)	Oasis MCX SPE (60 mg)	UHPLC-TQ-MS/MS	0.003–0.04	0.01–0.1	61–201	72–124	≤23.7	(15)
hot pot	hot pot (5 g)	MOR, COD, THEB, PAP, NOS	AcN 0.1% formic acid (20 mL, 10 min) and *n*-hexane (20 mL)	Fe_3_O_4_@SiO_2_@CS/GO (15 mg)	UHPLC-TQLIT-MS/MS	0.016–0.092	0.036–0.31	40–92	75–104	≤10	(27)
hot pot	hot pot (2 g)	MOR, COD, THEB, PAP, NOS	water/AcN 50% (20 mL, 5 min)	Fe_3_O_4_@SiO_2_@ADME (50 mg)	HPLC-TQLIT-MS/MS	0.05–0.8	0.25–2.5	76–80	80–115	≤10.7	(28)
breadsticks and sliced bread	breadsticks and sliced bread (2.5 g)	MOR, COD, THEB, PAP, NOS, ORIP	MeOH 0.1% acetic acid (10 mL, 30 min × 2)	Fe_3_O_4_@TPA–Fe (1 mg)	HPLC-TQ-MS/MS	0.3–13	1–42	64–120	70–110	≤20	this work

a MOR: morphine, COD: codeine, THEB: thebaine, PAP: papaverine, NOS: noscapine, NAR: narceine, ORIP: oripavine, Chl: chloroform, IPOH; isopropanol, AcN: acetonitrile, MeOH: methanol, HCl: hydrochloric acid, PE: petroleum ether, SPE: solid-phase extraction, MSPE: magnetic solid phase extraction, (U)HPLC: (ultra)-high-performance liquid chromatography, TQ: triple quadrupole, IT: ion trap, MS/MS: tandem mass spectrometry, TQLIT: triple quadrupole ion trap, MDL: method detection limit, MQL: method quantification limit, ME: matrix effect, RSD: relative standard deviation, and refs: references.

### Application of the Proposed Method to Real Samples of Bakery Products

The proposed method was applied to the analysis of nine bakery samples, five breadsticks, and four sliced bread (Table S1). To obtain the result of each sample, a range of concentrations obtained in the lowest and highest sample replicate are shown (Table 4). This is because the concentration that can be found in poppy seeds is highly variable, even in seeds from the same commercial batch,^2,6^ as the OA content depends on several factors such as climate, harvesting method, harvesting time, or plant variety.^14^ For this reason, each replicate (*n* = 6) is a proportion different form maybe different contamination. As shown in Table 4, the concentrations found in all breadsticks were low, only one of them could be quantified, thebaine, showing 0.22 ± 0.01 mg/kg (BS-4). Regarding the analytes identified, codeine was detected in one of them (BS-4), thebaine in three (BS-3, BS-4, and BS-5), papaverine in all except one (BS-5), noscapine in only one (BS-3), and morphine and oripavine were not detected in any of them. On the other hand, higher amounts were found in sliced bread samples (Table 4). Thus, morphine was found in two samples, with a maximum concentration of 0.16 ± 0.02 mg/kg (SB-4). For papaverine, all samples were below the MQL, except for one sample in which it was not detected (SB-4), noscapine was found in two samples (SB-1 and SB-3), where the highest concentration was 0.24 ± 0.01 mg/kg, and oripavine was not detected in any sample. In addition, considerably high levels for codeine and thebaine were found, which were identified in all samples, giving concentrations up to 8.3 ± 0.5 and 2.4 ± 0.2 mg/kg, respectively. Therefore, the poppy seeds used in the preparation of these products were highly contaminated. In this regard, considering that the average seed content in the product is 5%, the seeds would have approximately 166 mg/kg of codeine (SB-4) and 48 mg/kg of thebaine (SB-2). In addition, two sliced bread (SB-3 and SB-4) exceeded the EU maximum limit of 1.5 mg/kg morphine equivalents (morphine + 0.2 codeine) in the replicate with the highest amount. Comparing the results obtained with those of other authors on bakery samples, similar results were seen. Sproll et al. analyzed 12 samples of bread mix made with baked poppy seeds in which codeine, papaverine, and noscapine were not detected, and the morphine content found was between the MQL (<0.3 mg/kg) up to 4 mg/kg.^4^ López et al. analyzed two ready-to-eat bakery products (cakes) and found up to 0.6 mg/kg of morphine and <0.1 mg/kg of the remaining compounds.^3^ Carlin et al. in 2020 analyzed untreated poppy seeds, obtained considerable amounts of OAs, and then made muffins and bread coated with poppy seeds; they did not determine any OAs.^2^

**Table 4:** Range of Occurrence (mg/kg) ± SD (Standard Deviation) of the Six OAs in Six Replicates (*n* = 6) for Each of the Nine Bakery Products Analyzed^a^

sample code	morphine	codeine	thebaine	papaverine	noscapine	oripavine
BS-1	ND	ND	ND	<MQL	ND	ND
BS-2	ND	ND	ND	<MQL	ND	ND
BS-3	ND	ND	<MQL	<MQL	<MQL	ND
BS-4	ND	<MQL	<MQL to 0.22 ± 0.01	<MQL	ND	ND
BS-5	ND	ND	<MQL	ND	ND	ND
SB-1	ND	<MQL	<MQL	<MQL	<MQL	ND
SB-2	ND	<MQL to 1.03 ± 0.06	<MQL to 2.4 ± 0.2	<MQL	ND	ND
SB-3	<MQL to 0.09 ± 0.01	1.39 ± 0.08 to 7.4 ± 0.4	<MQL	<MQL	<MQL to 0.24 ± 0.01	ND
SB-4	<MQL to 0.16 ± 0.02	<MQL to 8.3 ± 0.5	<MQL	ND	ND	ND

a BS: breadsticks; SB: sliced bread; ND: not detected; and <MQL: lower than method quantification limit but higher than the method detection limit (MDL). SD: standard deviation calculated with the corresponding validation level at intraday precision.

Therefore, relatively low amounts of OAs were shown in this article as well as in other published articles. However, the OA levels were much lower than those obtained on poppy seeds in our previous work,^6^ where concentrations found were of up to 249 mg/kg morphine, 6 mg/kg codeine, 136 mg/kg thebaine, 27 mg/kg papaverine, 109 mg/kg noscapine, and 33 mg/kg oripavine. Estimating that these bakery products have 5% of seeds in their composition, it could be found up to 5% of these values previously found in seeds. For this reason, it could be suggested that the high temperatures used in the elaboration of these products may reduce the OA content, as reported in previous studies and in the recommendation of European Commission.^3,4,7,10,11,41^ Therefore, one might consider a higher decrease in breadsticks samples than in sliced bread samples, which may be attributed to the fact that in sliced bread, the poppy seeds are inside the bread, whereas in breadsticks, the poppy seeds are on the surface and the effect of heating is more pronounced. Hence, in the case of products that are subjected to high temperatures, like bakery products, a treatment should be established to ensure their reduction as much as possible. However, to confirm this factor, more studies should be carried out on how the food processing can interfere in different matrices and, especially, how it affects the other OAs that are also present in high concentrations and are even more potentially toxic than morphine and codeine.^8^

### Conclusions

An efficient, simple, and rapid method to quantify six OAs in bakery products with poppy seeds has been developed and validated for the first time. For this purpose, an SLE followed by MSPE purification using 1 mg of Fe_3_O_4_@TPA-Fe was performed in only 2 min and a posterior analysis by HPLC-MS/MS. The method was successfully validated with recovery values between 70 and 110%, RSD values ≤ 20%, and without matrix effects. The method was applied to nine bakery samples, five of them were breadsticks and four were sliced breads, showing lower amounts than poppy seeds, especially in breadsticks samples. However, two sliced bread samples exceeded the maximum level of new Commission Regulation (EU) 2021/2142. Therefore, in addition to morphine and codeine, further studies are needed on other OAs that may be even more toxic. In addition, it is necessary to study the possible effects of food processing to establish a treatment that guarantees their reduction as much as possible. In addition, the study of more types of samples to be able to legislate according to the levels of contamination is needed.

## Supporting Information Available

Different bakery products (breadsticks and sliced bread) analyzed in the present work to determine the concentration of six OAs; characterization of the Fe_3_O_4_@TPA–Fe material; method validation; SEM images of Fe_3_O_4_ and Fe_3_O_4_@TPA–Fe and TEM images of Fe_3_O_4_@TPA–Fe; ATR-FTIR spectrum of magnetite (Fe_3_O_4_), TPA, TPA–Fe, and Fe_3_O_4_@TPA–Fe materials; XRD patterns obtained for Fe_3_O_4_ and Fe_3_O_4_@TPA–Fe; N_2_ adsorption–desorption isotherms of Fe_3_O_4_ and Fe_3_O_4_@TPA–Fe; textural properties of materials synthesized; effect of pH on adsorption for each of the analytes at different times (1, 5, 10, 20, 30, and 60 min) with 2 mL of hexane with 10% formic acid, hexane, and hexane with 10% ammonia with 50 mg Fe_3_O_4_@TPA–Fe material; diagram of possible molecular interactions π–π, hydrogen bond, and ion–dipole between the adsorbent material and OAs (in the example, morphine); possible molecular interactions between the adsorbent material and OAs; equations of adsorption kinetics and isotherms; adsorption kinetic and isotherm experiments of the six OAs with 1 mg of Fe_3_O_4_@TPA–Fe material; kinetic parameters of the adsorption of six opioid alkaloids with 1 mg of Fe_3_O_4_@TPA–Fe material for different times (1–20 min) based on different kinetic models; three kinetics and two isotherm models for the adsorption of the six OAs with 1 mg of Fe_3_O_4_@TPA–Fe material; optimal parameters of MRM for the analysis of six OAs by HPLC–MS/MS; and instrumental validation parameters of the HPLC–MS/MS analysis (PDF)

## Abbreviations

OAs: opium alkaloids
SLE: solid–liquid extraction
MSPE: magnetic solid-phase extraction
Fe_3_O_4_@TPA–Fe: magnetite surface-modified with Fe(III) terephthalate
HPLC–MS/MS: high-performance liquid chromatography-tandem mass spectrometry
