# Supplementary material for: New Validated Method for the Determination of Six Opium Alkaloids in Poppy Seed-Containing Bakery Products by High-Performance Liquid Chromatography-Tandem Mass Spectrometry after Magnetic Solid-Phase Extraction
Source: J Agric Food Chem. 2022 Jun 8;70(24):7594–606. doi: 10.1021/acs.jafc.2c01664 (PMC9228061; doi:10.1021/acs.jafc.2c01664)
Supplement: Supplementary file 1 — jf2c01664_si_001.pdf [file jf2c01664_si_001.pdf]

Supporting Information

A new validated method for the determination of six opium alkaloids in poppy seed containing bakery products by high-performance liquid chromatography-tandem mass spectrometry after magnetic solid-phase extraction

Gema Casado-Hidalgo, Gonzalo Martínez-García, Sonia Morante-Zarcero, Damián Pérez-Quintanilla, Isabel Sierra*

Departamento de Tecnología Química y Ambiental, E.S.C.E.T, Universidad Rey Juan Carlos,

C/ Tulipán s/n, 28933 Móstoles, Madrid, Spain

* Corresponding author: Tel.: (+34) 914887018; fax: (+34) 914888143.

E-mail address: isabel.sierra@urjc.es

Table S1. Different bakery products (breadsticks and sliced bread) analysed in the present work to determine the concentration of six OAs.

Bakery product type	Code	Poppy seeds amount (%)
Breadsticks	BS-01	6
	BS-02	6
	BS-03	Not specified
	BS-04	Not specified
	BS-05	1
Sliced bread	SB-01	2.3
	SB-02	5
	SB-03	6
	SB-04	3

BS: breadsticks; SB: sliced bread. Poppy seed contents were obtained from the ingredients list on the product packages.

Supporting information S1. Characterization of the Fe_3O_4@TPA-Fe material.

Scanning electron microscopy (SEM) images were scanned by a Nova Nano SEM230 (FEG-SEM) (Denton, USA) with an energy-dispersive spectrometry system (EDS). The metalliser used was Leica ACE600 (Wetzlar, Alemania), using a gold target and depositing a layer of less than 5 nm. Transmission electron microscopy (TEM) images were performed on JEOL JEM 1010 (Tokyo, Japan) with an accelerating voltage of 80kV. The samples were dispersed in acetone and deposited on carbon-supported grids. Attenuated Total Reflection Fourier-Transform Infrared (ATR-FT-IR) spectra were recorded with a Spotlight 200i, Perkin Elmer (USA) spectrometer in the region 4000-400 cm^{-1}. Powder X-ray diffraction (XRD) patterns were recorded on a Philips Diffractometer model PW3040/00 X'Pert MDP/MRD (Eindhoven, Netherlands) at 45 kV and 40 mA by Cu Kα radiation (λ = 1.5418 Å). Nitrogen gas adsorption-desorption isotherms were performed with a Micromeritics ASAP 2020 (Norcross, USA) analyser. The isotherms were measured at -196 ˚C with an interval of relative pressures (P/P_0) from 10^{-4} to 0.994. Previously, samples were degassed at 80 ˚C under vacuum for 10 h. The Brunauer-Emmett-Teller (BET) method was utilized to obtain the specific surface area (S_{BET}), the Barrett-Joyner-Halenda (BJH) model was used to calculate the pore volume and pore size distribution by the desorption branches of isotherms and the total pore volume (V_t) was estimated from the desorbed amount at a relative pressure P/P_0 of 0.97. Finally, elemental analysis (% C and % N) was performed using a microanalyser Flash 2000 Thermo Fisher Scientific Inc. (Hampton, USA).

Supporting information S2. Method validation.

Linearity was assessed with matrix-matched calibration curves prepared in three consecutive days. All these curves were prepared for blank breadsticks (BS-1) and sliced bread (SB-1) samples at six known concentration levels within the linear range evaluated. For this purpose, the sample extracts obtained after the SLE-MSPE procedure were spiked with an aliquot of a standard solution containing the target alkaloids according to the desired concentration level of the calibration curve. In addition, quantification of morphine by means of isotope labelled IS correction was carried out. To do this, 50 µL of 1 µg/mL IS were added to each point of the matrix-matched calibration curves. The criteria for good linearity involve values ≤ ± 20% for the deviation of the back-calculated concentrations of the calibration standards from the true concentrations. Matrix effects were determined by comparing the slopes of the calibration equations obtained from both matrix-matched and solvent-based calibration curves (both expressed in the same units µg/mL), calculating (1 - the ratio slope matrix-matched/slope solvent-based) × 100 for each analyte. The ME is lower when closer to 0% and according with the guideless the ME is negligible when is lower than ±20%. Positive values greater than 20% indicate signal enhancement and negative values indicate signal suppression. However, when the signal suppression or enhancement is greater than this margin of 20%, matrix effects must be considered in calibration. The sensitivity of the method for each sample was determined through the MDLs and MQLs of the OAs from the analysis of the lowest concentration analysed (0.01 or 0.001 µg/mL), which were estimated as the minimum concentration yielding a signal-to-noise ratio (S/N) of 3 or 10, respectively. The recovery assays were assessed by comparing the areas obtained for samples spiked (n = 6) with a known concentration of analytes and subjected to the SLE-MSPE procedure with those areas obtained for simulated samples (samples spiked at the same concentration but at the

end of the procedure prior to their chromatographic analysis). The recovery assays were performed spiking the bread samples at a concentration of 5 mg/kg (high value) and 0.25 mg/kg (low value). According to the validation guidelines, the recovery values should be between 70 and 120%. On the other hand, the method precision was evaluated in terms of repeatability and reproducibility, using the same validation levels (low and high) as for the accuracy. For repeatability (expressed as RSD%), a sample spiked with the OAs at the corresponding validation level was consecutively carried out six times (n = 6) on the same day. The reproducibility (also expressed as RSD %) was calculated by the analysis of three replicates of a sample (spiked with the analytes at the corresponding validation level), which were carried out in triplicate through three different days (n = 9). According to the validation guidelines, the RSD values for these precision parameters should be ≤ 20%. The selectivity of the method was determined by comparing the spectra of the different analytes obtained from standard solutions with the spectra obtained in the samples. It was considered satisfactory when the variation in the spectra was less than ± 30 % and the retention time of the target analytes was within the interval ± 2.5 %.

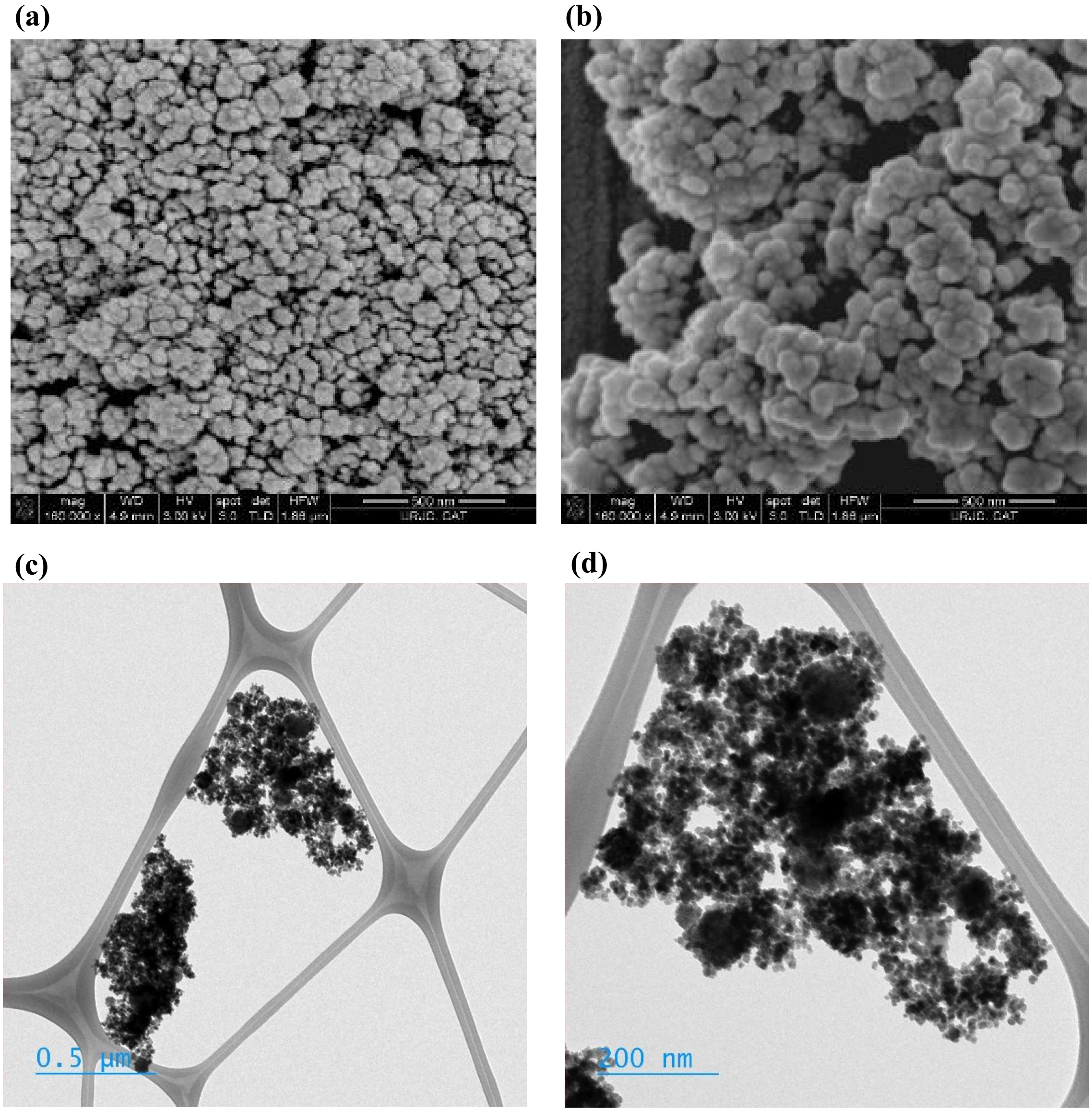

Fig. S1. SEM images of Fe_3O_4 (a) and Fe_3O_4@TPA-Fe (b) and TEM images of Fe_3O_4@TPA-Fe (c and d).

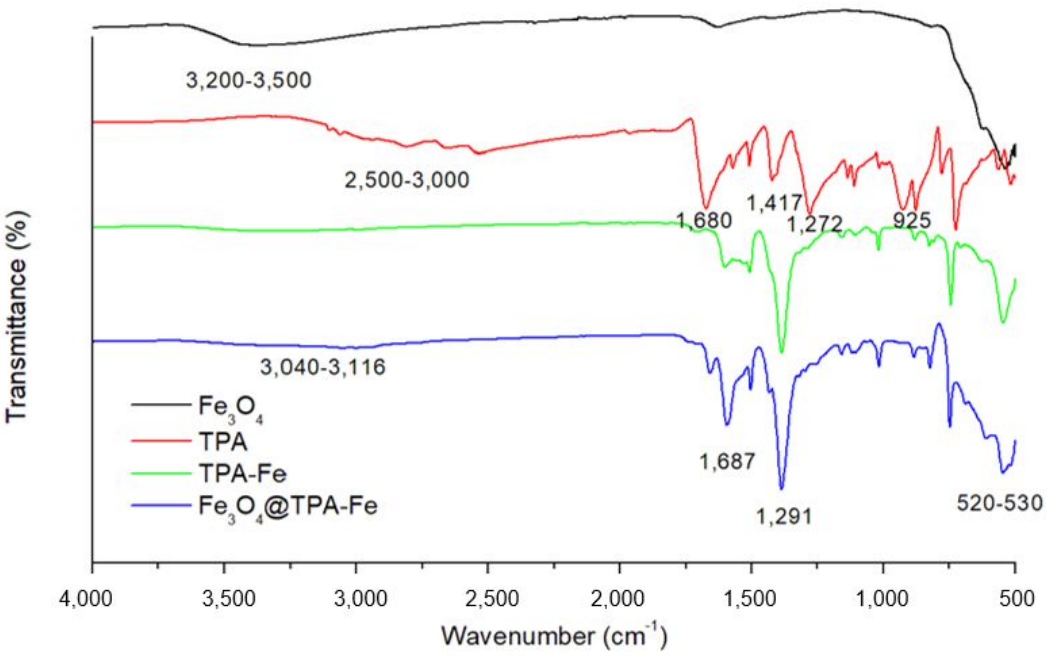

Fig. S2. ATR-FTIR spectrum of magnetite (Fe_3O_4), terephthalic acid (TPA), TPA-Fe and Fe_3O_4@TPA-Fe materials.

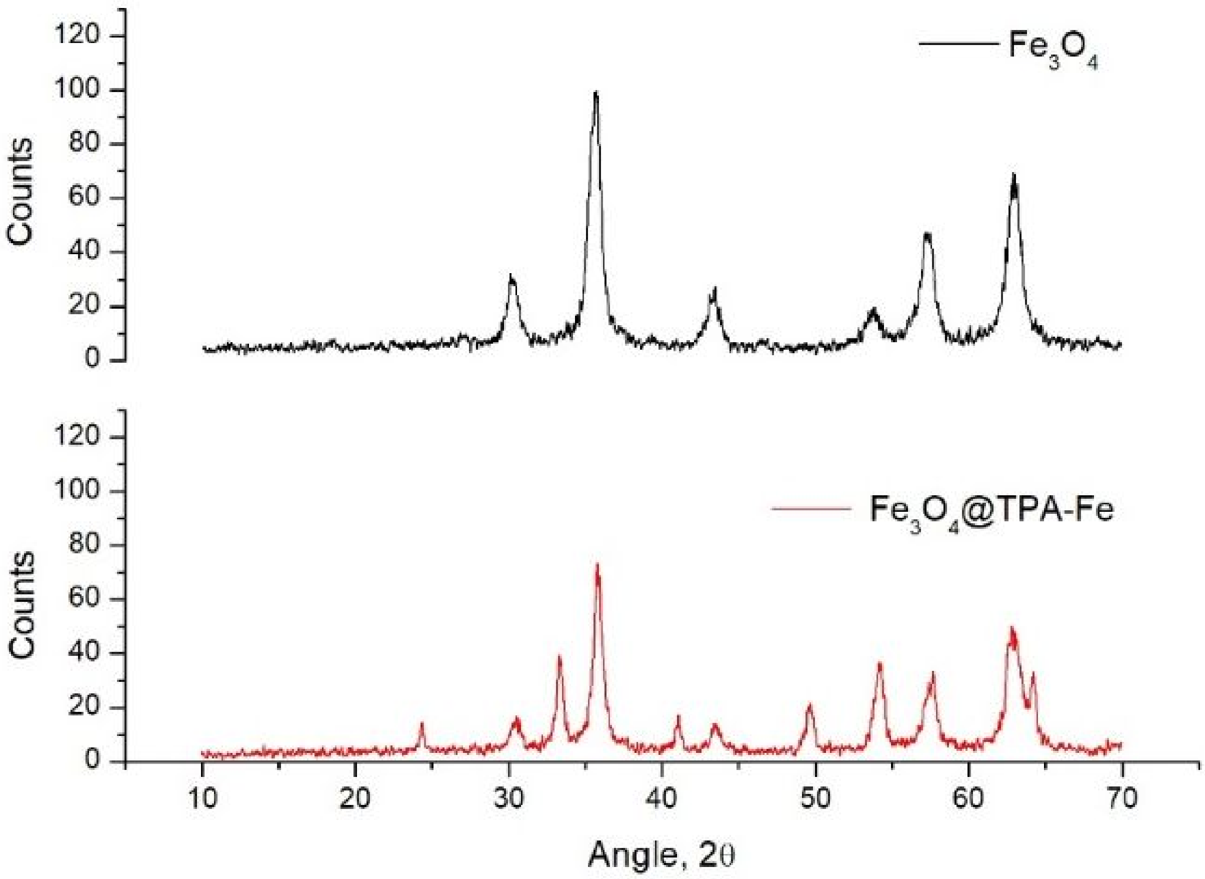

Fig. S3. XRD patterns obtained for Fe_3O_4 and Fe_3O_4@TPA-Fe.

(a)

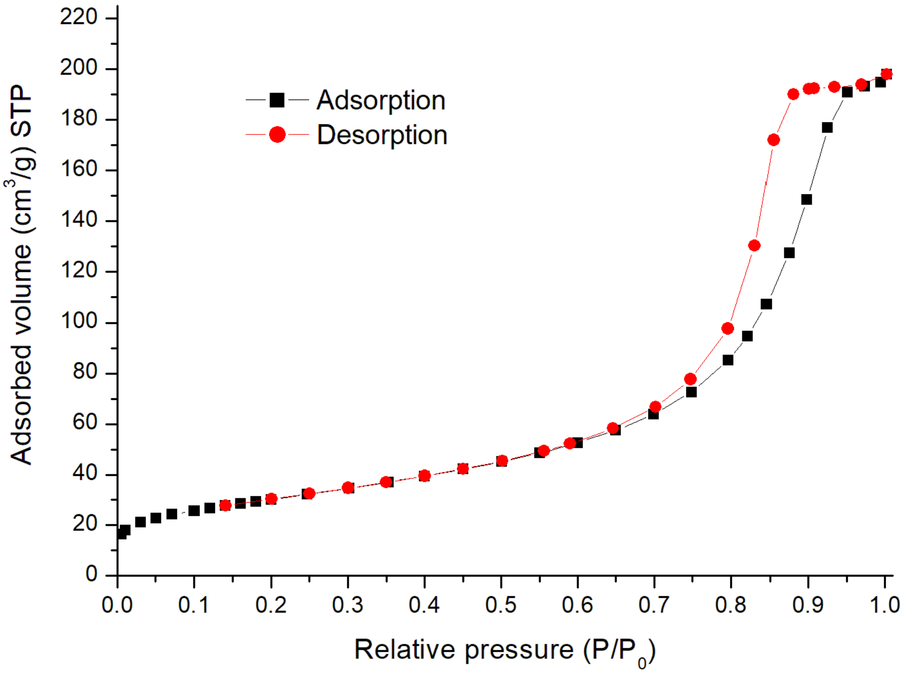

(b)

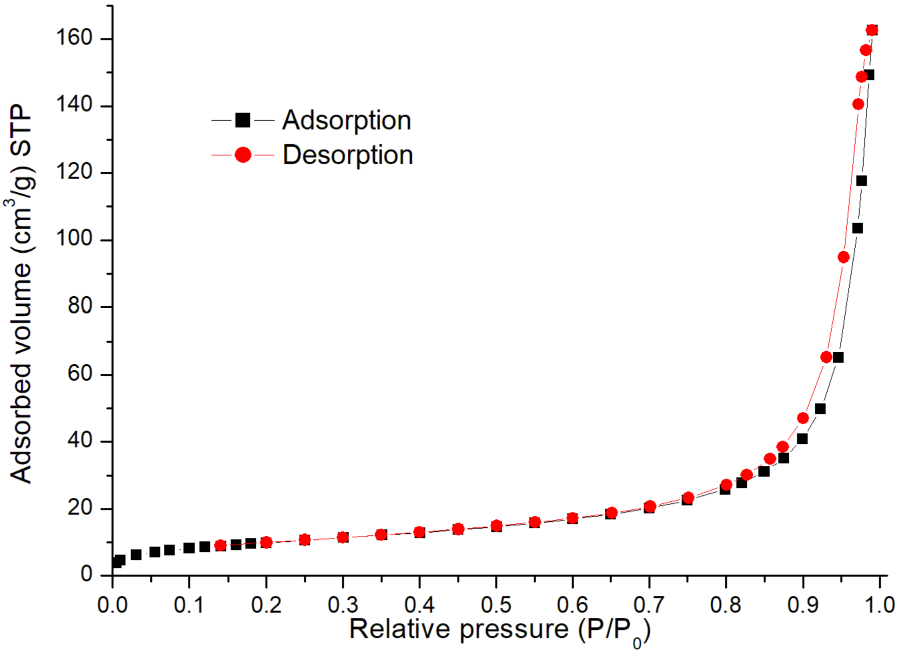

Fig. S4. N_2 adsorption-desorption isotherms of Fe_3O_4 (a) and Fe_3O_4@TPA-Fe (b). STP: standard temperature and pressure.

Table S2. Textural properties of materials synthesized.

Material	S_{BET} (m2/g)[a]	Pore volume (cm^3/g)[b]	Pore diameter (Å)[c]
Fe_3O_4	105	0.30	41.3 and 130.1
TPA-Fe	718	0.50	20.8, 92.9 and 279.2
Fe_3O_4@TPA-Fe	47	0.14	21.0, 93.3, 131.8, 237.8 and 488.3

[a] S_{BET}: Specific surface area calculated by Brunauer-Emmett-Teller (BET) method.

[b] Total pore volume was measured at relative pressure (P/P_0) = 0.97.

[c] Pore diameter estimated by using the BJH (Barrett, Joyner and Halenda) model applied on the desorption Branch.

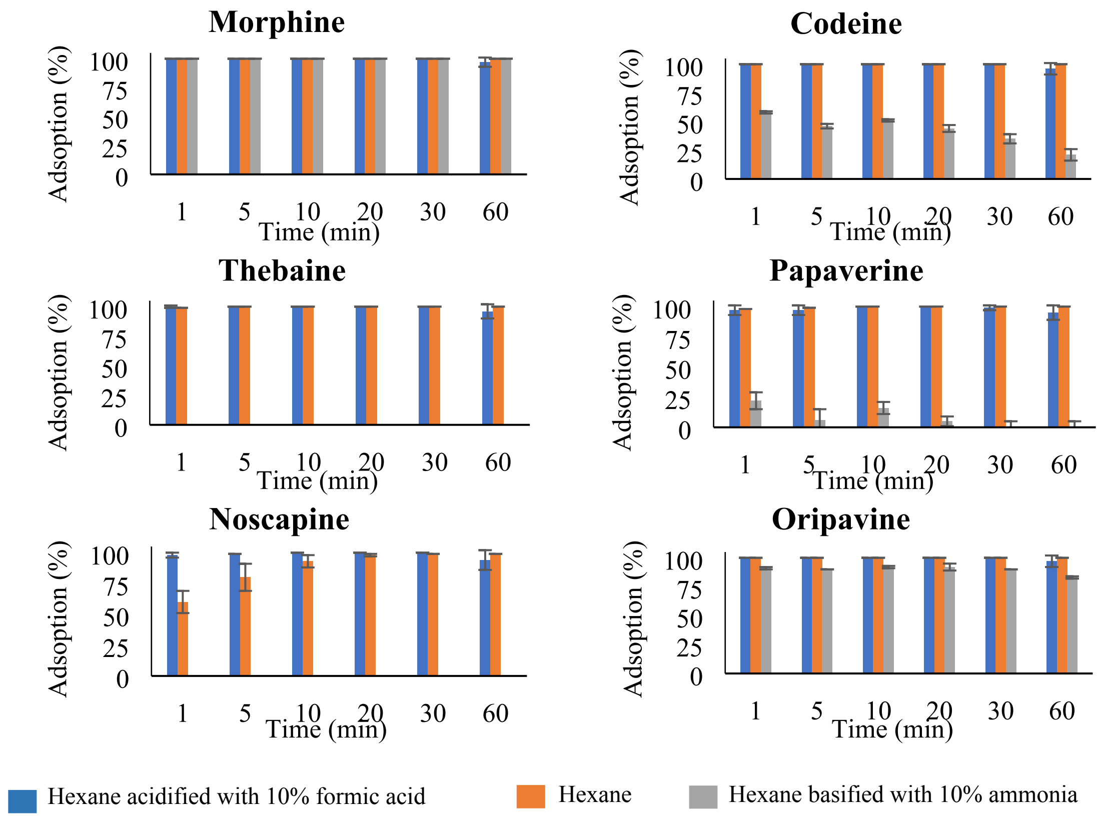

Fig. S5. Effect of pH on adsorption for each of the analytes at different times (1, 5, 10, 20, 30 and 60 min) with 2 mL of hexane with 10% formic acid, hexane and hexane with 10% ammonia with 50 mg Fe_3O_4@TPA-Fe material.

(a) **π-π Electrostatic interaction**

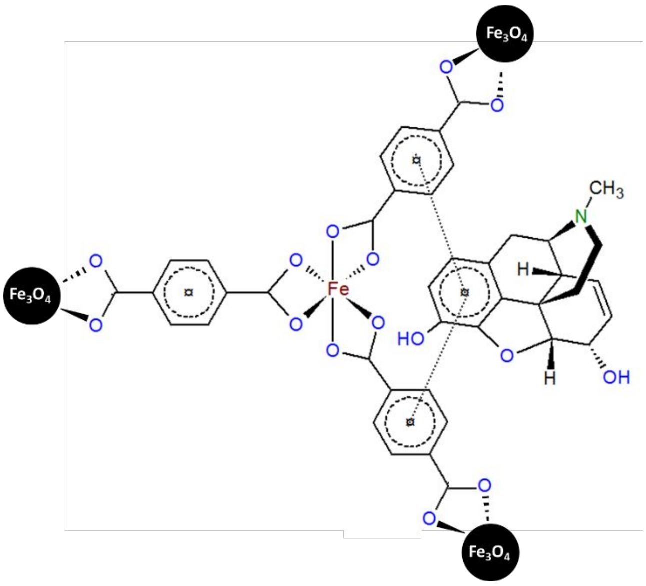

(b) **Hydrogen bond interaction**

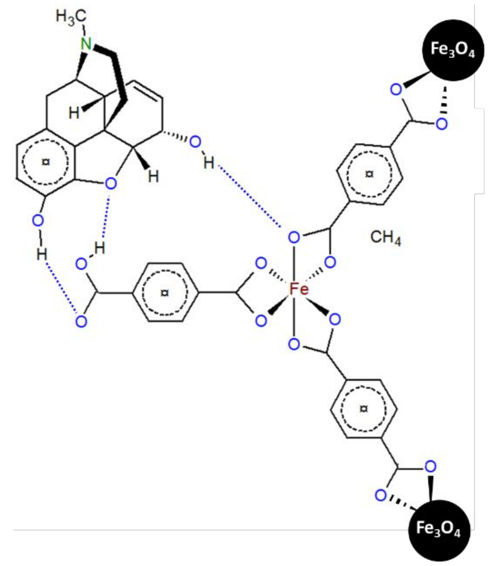

(c) **Ion-dipole interaction**

(Favoured in acidified medium)

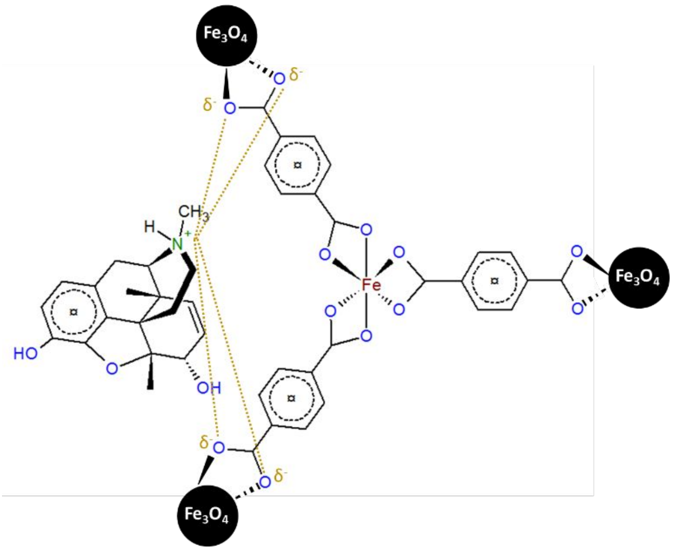

Fig. S6. Diagram of possible molecular interactions π-π (a), hydrogen bond (b) and ion-dipole (c) between the adsorbent material and OAs (in the example, morphine).

Supporting information S.3. Possible molecular interactions between the adsorbent material and OAs.

As OAs have a structure composed of aromatic rings, possible electrostatic interactions were considered, especially π-π, between the π cloud of the opium alkaloid and that of the TPA (Fig. S6a). In addition, hydrogen bonds between the -OH of the analytes and the acids groups of the material were expected (Fig. S6b). Besides, with acidic pH, the nitrogen of the amino groups of the alkaloids can be protonated giving a positive charge that interacts ionically with the polar group of the TPA (Fig. S6c). This last interaction was important for noscapine as it allowed 100% adsorption in the first minute when the adsorption medium was acidified.

Table S3. Equations of adsorption kinetics and isotherms

$Q_e = \frac{(C_o - C_e)V}{W}$	Adsorption capacity	(1)
$\ln(q_e - q_t) = lnqe - k_1 t$	Lagergren's pseudo-first order	(2)
$\frac{t}{q_t} = \frac{1}{k_2 q_e^2} + \left[\frac{1}{q_e}\right] t$	Pseudo-second order	(3)
$q_t = k_p t^{1/2} + C$	Intra-particle diffusion	(4)
$\frac{1}{Q_e} = \frac{1}{Q_{max}} + \frac{1}{K_L Q_{max} C_e}$	Langmuir model	(5)
$Log\ q_e = logK_F + \frac{1}{n} logC_e$	Freundlich model	(6)

C_o and C_e: initial and equilibrium concentrations of the target analytes (µg/L), respectively; V: volume of the solution (L); W: mass of the adsorbent (g); k_1: pseudo-first order rate constant (min^{-1}); q_e and q_t: amounts of OAs adsorbed at equilibrium and time (mg/g), respectively; k_2: pseudo-second order adsorption rate constant (g/mg min); q_t: amount of OAs adsorbed at time t (mg/g); k_p: intraparticle diffusion rate (mg/L min^2); C: intercept; Q_{max}: maximum monolayer capacity of the adsorbent (mg/g); K_L: Langmuir binding constant which is related to the energy of adsorption (L/mg); K_F: is the Freundlich constant (L/mg); n: is the heterogeneity factor (dimensionless).

(a)

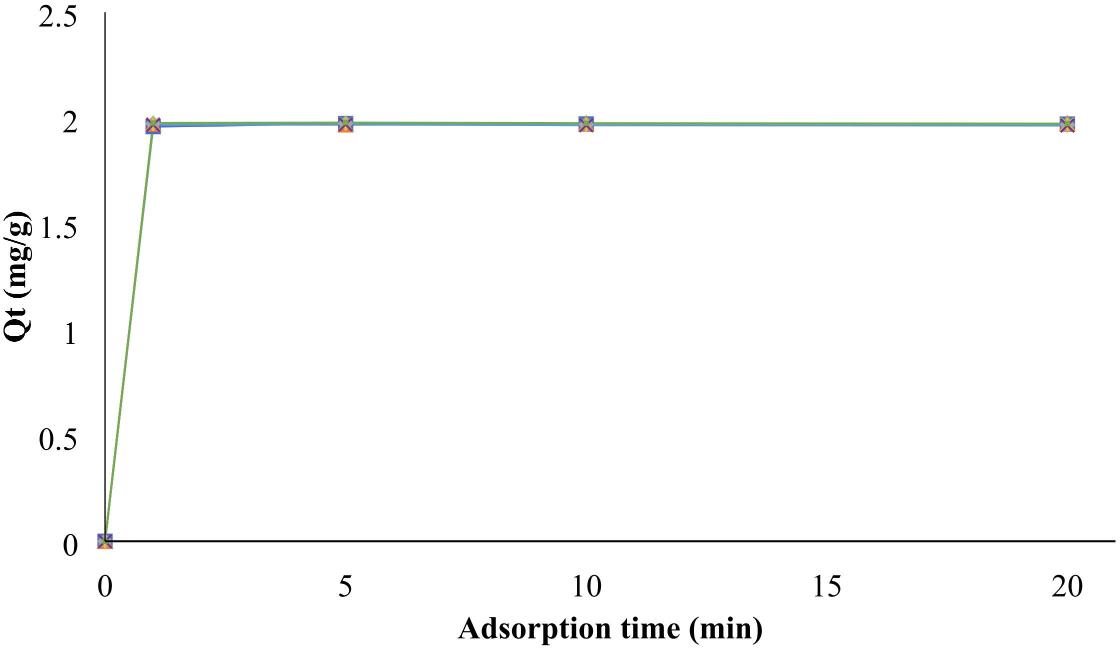

(b)

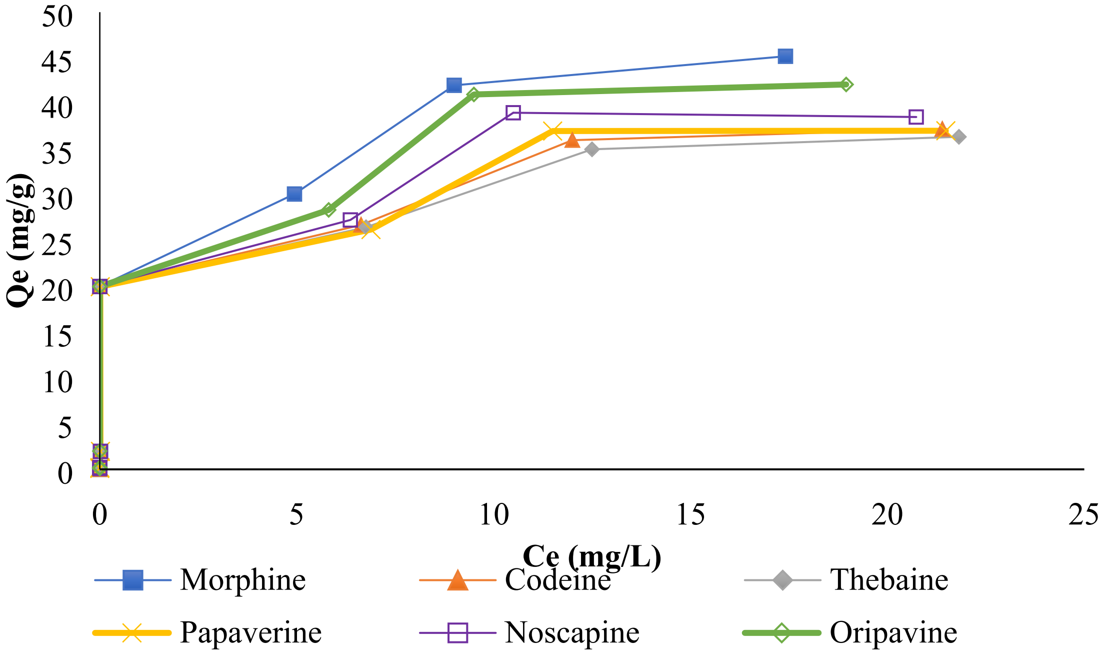

Fig. S7. Adsorption kinetic (a) and isotherm (b) experiments of the six OAs with 1 mg of Fe_3O_4@TPA-Fe material. Qe and Qt: amounts of OAs adsorbed at equilibrium and time (mg/g), respectively; Ce: initial and equilibrium concentrations of the target analytes (μg/L).

Table S4. Kinetic parameters of the adsorption of six opioid alkaloids with 1 mg Fe_3O_4@TPA-Fe material for different times (1-20 min) based on different kinetic models.

		**Pseudo-first order model**			**Pseudo-second order model**			**Intra-particle model**		
	**$Q_{e, exp}$ (mg/g)**	**R^2**	**K_1 (min^{-1})**	**$Q_{e, cal}$ (mg/g)**	**R^2**	**K_2 (g/mg min)**	**$Q_{e, cal}$ (mg/g)**	**R^2**	**K_p (mg/g min^2)**	**C**
Morphine	1.96	0.018	-0.061	0.028	1	5804	1.703	0.472	0.003	1.961
Codeine	1.97	0.031	0.070	0.018	1	1344	1.972	0.278	0.001	1.969
Thebaine	1.97	0.799	0.378	5.312	1	758	1.968	0.707	-0.002	1.976
Papaverine	1.97	0.799	0.312	3.975	1	1292	1.968	0.256	-0.001	1.972
Noscapine	1.97	0.671	0.343	1.116	1	1015	1.965	0.575	-0.001	1.970
Oripavine	2.00	0.570	-0.008	0.022	1	1053	1.972	0.476	-0.001	1.979

$Q_{e, exp}$: amounts of OAs adsorbed at equilibrium, experimental; K_1: pseudo-first order rate constant constant; $Q_{e, cal}$: amounts of OAs adsorbed at equilibrium, calculated; K_2: pseudo-second order adsorption rate constant; K_p: intraparticle diffusion rate; C: intercept.

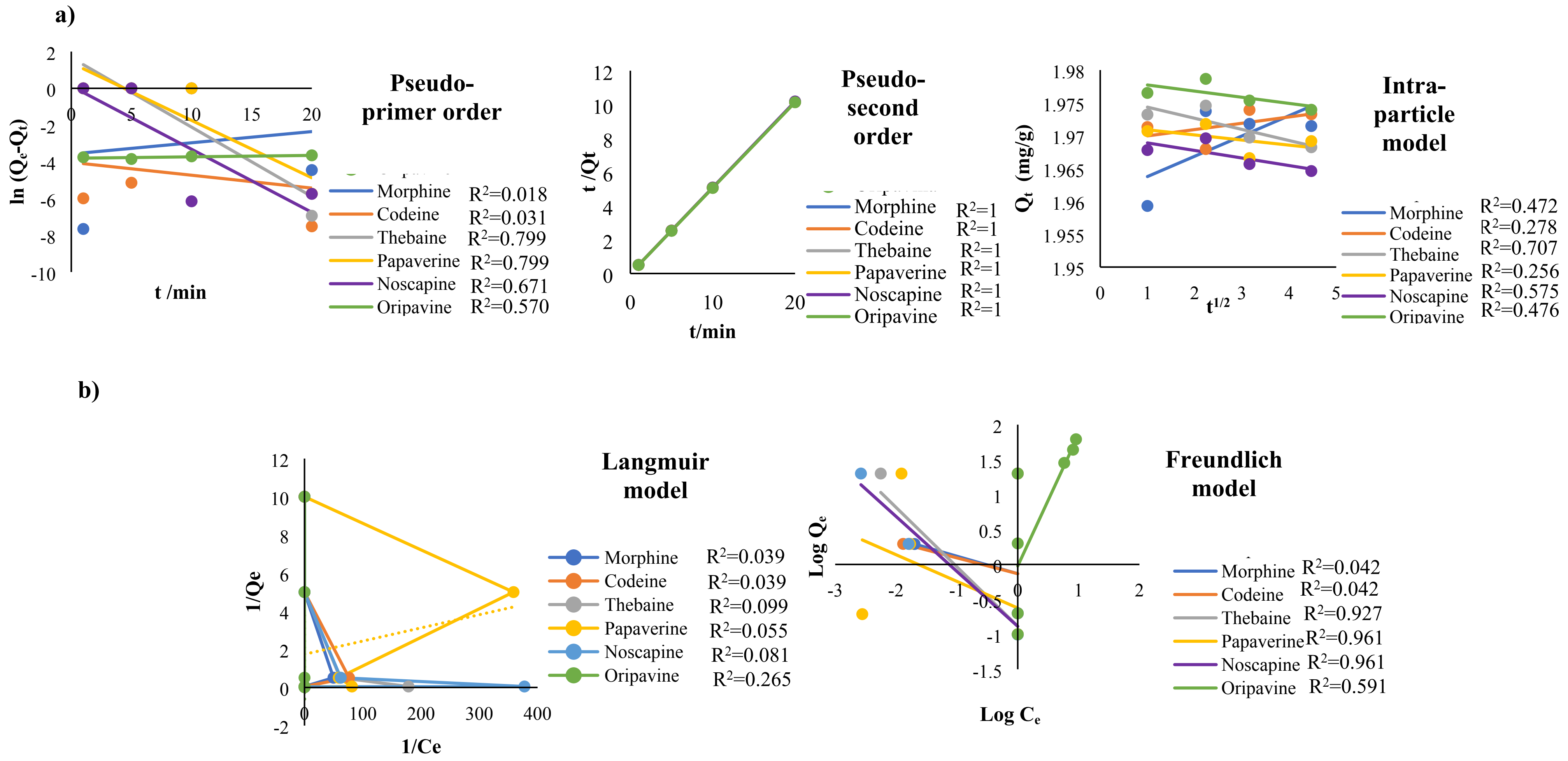

Fig. S8. Three kinetics (a) and two isotherm models (b) for the adsorption of the six OAs with 1 mg of Fe_3O_4@TPA-Fe material. Qe and Qt: amounts of OAs adsorbed at equilibrium and time (mg/g), respectively; Ce: initial and equilibrium concentrations of the target analytes (μg/L).

Table S5. Optimal parameters of MRM for the analysis of six OAs by HPLC-MS/MS.

Analytes	t_R[a] (min)	Ionization mode	Precursor ion (Q_1, m/z, $[M+H]^+$)	Fragment ion[b] (Q_3, m/z)	CV[c] (V)	CE[d] (eV)
Morphine	5.778	ESI (+)	286.1	**153.0**	72	45
				165.0		24
				228.6		22
Morphine-d3	5.819	ESI (+)	288.7	**152.3**	72	45
				164.2		37
				200.6		25
Codeine	5.869	ESI (+)	300.2	153.1	72	45
				165.0		45
				**215.1**		24
Oripavine	6.020	ESI (+)	298.3	236.9	72	14
				**249.1**		17
				267.1		12
Thebaine	6.245	ESI (+)	312.3	**58.2**	72	8
				249.4		16
				166.2		16
Papaverine	6.270	ESI (+)	340.2	**202.0**	72	24
				324.1		30
Noscapine	6.303	ESI (+)	414.3	179.0	72	24
				205.1		42
				**220.0**		20

[a] t_R: retention time; [b]: the fragment ion used for the quantification are in bold. Gradient elution with a mobile phase of acetonitrile (A) and water (B), both with 0.1% of formic acid. The gradient started at 90% B, in min 6 changed to 30% B, in min 9 returned to 90% B and it was maintained until min 11 to equilibrate.

[b] The quantitation ion transitions are in bold and two transitions used for the confirmation of detection are underlined.

[c] CV: cone voltage.

[d] CE: collision energy.

Table S6. Instrumental validation parameters of HPLC-MS/MS analysis.

Instrumental validation				
**Analytes**	**Linear range (μg/mL)**	**Solvent calibration (R^2)**	**LOQ (μg/L)**	**LOD (μg/L)**
Morphine	0.01-1	$y = 3.6\times10^5x + 2.3\times10^4$ (1.000)	3	1
Codeine	0.01-1	$y = 4.2\times10^6x + 1.9\times10^4$ (1.000)	5	1.5
Thebaine	0.001-1	$y = 4.0\times10^7x + 5.4\times10^5$ (1.000)	0.3	0.1
Papaverine	0.001-1	$y = 5.5\times10^7x + 7.6\times10^5$ (1.000)	0.1	0.04
Noscapine	0.001-1	$y = 7.7\times10^7x + 1.0\times10^6$ (0.999)	0.1	0.06
Oripavine	0.01-1	$y = 5.3\times10^6x + 2.4\times10^4$ (1.000)	6	1

LOQ: limit of quantification; LOD: limit of detection.
